# Allosteric Interactions of Flavonoids with ACE2 and Potential Implications for COVID-19: Computational Insights, Metabolic and Mitochondrial Effects of Rutin in A549 Cells

**DOI:** 10.1007/s12013-026-02093-1

**Published:** 2026-06-08

**Authors:** Fave Y. Tata, Mthokozisi B. Nxumalo, Vincent A. Obakachi, Hezekiel M. Kumalo, René B. Khan

**Affiliations:** 1 Drug Research and Innovation Unit, Discipline of Medical Biochemistry, School of Laboratory Medicine and Medical Science, University of KwaZulu-Natal, Durban, 4000 South Africa; 2 Molecular and Clinical Pharmacology Research Laboratory, Department of Pharmacology, Discipline of Pharmaceutical Sciences, University of KwaZulu-Natal, P.O. Box X5401, Durban, 4000 South Africa; 3 Department of Pharmaceutical Chemistry, Discipline of Pharmaceutical Sciences, College of Health Sciences, University of KwaZulu-Natal, Durban, South Africa

**Keywords:** ACE2, COVID-19, Flavonoids, Cytotoxicity, Rutin, Molecular dynamic simulations

## Abstract

There has been a compelling need to identify new therapeutic targets and agents owing to the increasing complexity of disease mechanisms. Angiotensin-converting enzyme 2 (ACE2), the primary host receptor for severe acute respiratory syndrome coronavirus-2 (SARS-CoV-2, plays a crucial role in the pathophysiology of COVID-19, making ACE2 a potential biological target. Identification of small molecules that can interact with ACE2 may offer insights into a host-targeted approach relevant to viral entry and ACE2-associated cellular pathways. The study evaluated allosteric interactions of selected flavonoids with ACE2, the cytotoxicity and metabolic effects of the promising candidate in A549 cells through computational and in vitro methods. Molecular docking identified flavonoid binding to ACE2, followed by a 120 ns molecular dynamics (MD) simulation to characterize the stability and dynamic behaviour of ACE2-flavonoid complexes. MM/GBSA binding free energy calculations and per residue energy decomposition were performed to identify key interactions stabilizing ligand binding. The in silico analyses showed ARG255, PRO328, GLU357, GLU384, GLU388, and ARG500 as the key allosteric residues contributing to ACE2-flavonoid complex stability. Rutin exhibited the most favourable binding affinity, followed by isoquercetin. Based on computational findings, rutin was selected for evaluation in A549 cells to assess cytotoxicity, metabolic and mitochondrial effects using MTT assay, ATP quantification, mitochondrial membrane potential (MMP), CYP3A4 activity and LDH release for membrane integrity. In vitro analyses showed that rutin is non-cytotoxic up to 500µM, enhances ATP production without adversely affecting MMP and membrane integrity. These findings indicate that rutin formed stable interactions with ACE2, with the potential to influence cellular energy metabolism without inducing cytotoxicity and therapeutic benefits in conditions linked to mitochondrial dysfunction and metabolic stress. While direct ACE2 enzymatic modulation was not confirmed experimentally, these findings provide a mechanistic basis for further studies on ACE2-flavonoid interactions and their potential biological implications in ACE2-associated diseases.

## Introduction

 The search for new therapeutics to treat diseases and emerging health conditions has been in the spotlight. Particularly, the recent emergence of the COVID-19 pandemic has sparked significant interest in finding effective therapeutic agents. Several therapeutic options have been explored to combat the high infectivity, mortality rate, and complex pathophysiology of COVID-19. Both viral and host components have been targeted to identify effective treatments and reduce infectivity [1–3]. SARS-CoV-2 proteins such as the main protease (Mpro), RNA-dependent RNA polymerase (RdRP), and spike proteins have been extensively studied [4–7], with comparatively less focus has been placed on the host angiotensin-converting enzyme 2 (ACE2), the critical receptor for viral entry, largely due to concerns over disrupting its vital physiological functions [8]. ACE2 plays useful physiological roles in the renin-angiotensin-aldosterone system (RAAS), where it counteracts the harmful effects of ACE by converting angiotensin II (Ang II) to angiotensin 1–7 (Ang 1–7), a vasodilatory peptide with anti-inflammatory and cardioprotective effects [8, 9]. Through this mechanism, ACE2 prevents vasoconstriction, oxidative stress, pro-inflammatory cytokine release, epithelial cell apoptosis, and insulin resistance [9–11], hence essential for maintaining cardiometabolic and pulmonary homeostasis. The SARS-CoV-2 has broad tissue tropism, binding to ACE2 through its spike protein, facilitating its invasion into host cells and pathogenicity [12, 13]. ACE2 is widely distributed and expressed in various tissues, including the gastrointestinal tract, brain, and alveolar epithelial cells of the lung [9, 14], rendering these tissues, particularly the lungs, highly vulnerable to viral infection and subsequent tissue damage. The binding of SARS-CoV-2 to ACE2 disrupts its functions [15], leading to immense immune responses, hyperinflammation and lung injury, contributing to the severity of COVID-19, which was a major factor in COVID-19 morbidity and mortality [16–18]. ACE2 has protective effects on pulmonary vascular circuits and reduces the progression of severe pneumonia and lung injury, highlighting its importance in lung health [19–21]. Phosphorylation of ACE2 at residue Ser680 by AMPK has been reported to enhance its stability and function in converting Ang II to the vasodilatory peptide Ang 1–7. This activates endothelial nitric oxide synthase (eNOS), which promotes nitric oxide (NO) bioavailability and vasodilation [22]. The convergence of metabolic [23–26] and infectious pathologies in COVID-19, particularly in patients with comorbidities such as diabetes and cardiovascular diseases [27, 28], underscores the need to explore ACE2 as a therapeutic target for viral and cardiometabolic diseases.

**Fig. 1:**
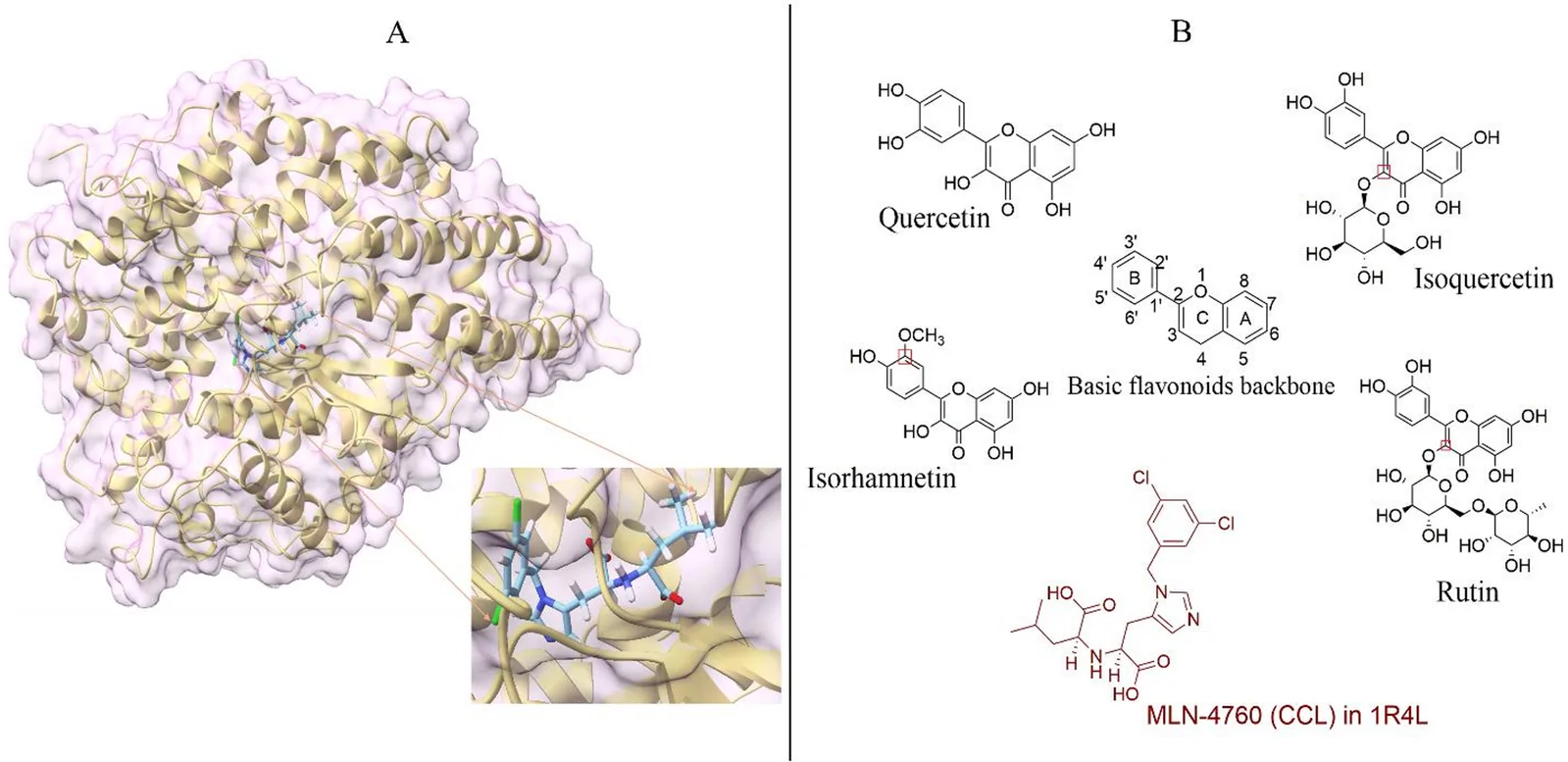
Illustrates: **A** The 3-D surface interaction of the angiotensin-converting enzyme 2 (ACE2) receptor with the co-crystallized ligand (CCL), **B** The basic flavonoid backbone, the flavonoids, and the CCL

Flavonoids, plant-derived bioactive molecules with antioxidant, anti-inflammatory, anticancer, antiviral and neuroprotective properties [29, 30], have demonstrated therapeutic potentials against various diseases, including diabetes and COVID-19 [30, 31]. Many preclinical studies have investigated flavonoids targeting SARS-CoV-2 viral proteins. However, there are limited studies on their interaction with the host ACE2 receptor. Elucidating ACE2-flavonoid molecular interactions is pivotal in understanding their therapeutic potential in modulating ACE2 to inhibit viral entry mechanisms while promoting ACE2-mediated cardiometabolic homeostasis. Interestingly, Recent reports have indicated the potential inhibitory effects of flavonoids against SARS-CoV-2 proteins [32, 33] and their protective effects against oxidative stress in diabetic animal models through ACE2-related pathways [34, 35]. Additional in vitro studies demonstrated their potential to ameliorate complications related to COVID-19 and diabetes [4, 36]. However, their exact mechanisms of action with most protein targets are not well understood. Molecular interactions and mechanistic insights from cells and animal models are often not explicit and well understood. Hence, in silico methods such as molecular docking, molecular dynamics and MM/GBSA binding free energy analyses have been utilized to characterize protein-ligand interactions, identify and prioritize flavonoid candidates with high binding affinity and specificity to ACE2 (Fig. 1A and B). Assessing the safety and metabolic effects of flavonoids is essential for their development as effective treatments, as this has limited clinical translation of potential therapeutic interventions. This study combined computational methods with in vitro cytotoxicity and biochemical assays to evaluate the binding affinity and stability of selected flavonoids with ACE2, followed by the safety and cellular effects of the prioritized flavonoid candidate.

## Materials and Methods

### Preparation of Protein and Ligands

The 3D protein structure of the ACE2 enzyme (PDB code: 1R4L, Fig. 1A) was obtained from the protein data bank [37], and the ligands (Fig. 1B) were obtained from PubChem, verified and cleaned using ChemDraw Ultra v14.0. The protein structure and the ligands were processed and optimized for molecular docking studies using UCSF Chimera v1.16 and ChimeraX [38], which involved deleting water molecules and non-essential heteroatoms, adding hydrogen atoms, and assigning appropriate atomic charges using Gasteiger by running dock prep. The 3D conformations of the ligands (Fig. 1B) were generated and processed in UCSF Chimera v1.16, where all hydrogen atoms were added and assigned appropriate charges to set the protonation state at pH 7.4. Ligand geometry optimization and energy minimization were carried out in Avogadro using the MMFF94 force field, and applying a convergence threshold of 10⁻⁶ kcal/mol to obtain energetically stable conformations [38, 39].

### Molecular Docking Screening

The molecular docking study utilized UCSF Chimera version 1.16 for the analysis. Autodock Vina was employed to generate the receptor grid and calculate the binding center and dimensions [40, 41]. A grid box was positioned at x = 39.48, y = 5.79, and z = − 28.30 with the side lengths of 14.42, 7.50, and 13.90, respectively, centred at the receptor’s binding site. The ligands were then docked into the ACE2 binding site, and the docking scores were evaluated to identify the optimal docking poses for each ligand. The ligands were ranked based on their docking scores, and the top 4 docked flavonoids with the highest scores for each ligand were selected for molecular dynamics (MD) simulations to assess the stability and dynamics of the protein-ligand complexes. The docked compounds were visualized using ChimeraX and Discovery Studio Visualizer v21.1.0.20298 [39, 42].

### Molecular Dynamic Simulations

The molecular dynamics (MD) simulations were performed following previously described protocols [43, 44]. MD simulations were conducted for 120 nanoseconds (ns) using a graphics processing unit (GPU) version of the Particle Mesh Ewald Molecular Dynamics (PMEMD) module in Amber 18 [45]. The Amber FF14SB force field was used for force field parameters and protein description. Ligand parameters were assigned using Gasteiger charges in Avogadro and the Antechamber module with the Generalized Amber Force Field (GAFF) [46, 47]. Hydrogen atoms were added to the receptor and counter ions for system neutralization using the LEAP module in Amber14 [48]. The protein-ligand complex was solvated in a TIP3P water box with a 10 Å distance between the protein surface and the box boundary [48]. Periodic boundary conditions were utilized and long-range electrostatic interactions were computed using PMEMD in Amber18 with a 12 Å Van der Waals cut-off [49]. Energy minimization was carried out using the steepest descent algorithm with a restraint potential for the initial minimization process set at 500 kcal/mol Å 2 for the solute for 1000 steps to relax the system, followed by a minimization without restraint to remove potential steric clashes. The system was gradually heated from 0 to 300 K for 300 ps with harmonic restraints of 5 kcal/mol Å^2^ for solute atoms at a constant volume and equilibrated under constant Number, pressure and temperature (NPT) conditions for 2 ns at 300 K and 1 bar pressure without restrictions using the Langevin thermostat. Electrostatic interactions were calculated using the Particle Mesh Ewald method and the SHAKE algorithm was applied to constrain bonds with hydrogen atoms [46, 50]. A 120 ns MD simulation production run was performed with a time step of 2 fs in an isothermal isobaric (NPT) ensemble, at 300 K and 1 bar pressure using Berendsen barostat with a 2 ps pressure-coupling constant. Trajectory coordinates were saved every 100 ps. Post-MD simulation analyses were carried out using the CPPTRAJ module of Amber14, including root mean square deviation (RMSD), root mean square fluctuations (RMSF), radius of gyration (RoG), principal component analysis (PCA), hydrogen bonds analysis, hydrogen bond occupancy and the binding free energy calculations performed using molecular mechanics/generalized-born surface area (MM/GBSA) using Amber18 [45, 51]. A total of 1000 evenly spaced frames were extracted at regular intervals from 20 ns to 120 ns for structural analyses. Principal component analysis (PCA) was computed using Cα trajectories from the 1000 equally spaced frames to evaluate dominant motion. Molecular structures were visualized using UCSF Chimera (version 1.16), and graphical plots were generated using Origin version 6.0 [52].

### Binding Free Energy Calculation

The MM/GBSA method in Amber18 was used to calculate the binding free energy, which was decomposed into van der Waals, electrostatic, polar and nonpolar solvation components to assess their respective contributions to the receptor-ligand complex interactions. This thermodynamic quantity was determined by analysing the energy difference between the protein-ligand complex and the unbound states of the protein and ligand. The MMGBSA method decomposes the binding energy into various components, including gas phase energy, solvation energy, and entropy [43]. Hence, the binding free energy is the sum of the molecular mechanics energy ($\:\varDelta\:{\mathrm{E}}_{\mathrm{g}\mathrm{a}\mathrm{s}}$), the entropy ($\:-\mathrm{T}\varDelta\:\mathrm{S}$) and the solvation free energy ($\:{\mathrm{G}}_{\mathrm{s}\mathrm{o}\mathrm{l}}$) as per the equation detailed in [43, 44]. The method decomposes the binding free energy of a system according to:

1 $$\:\varDelta\:{\mathrm{G}}_{\mathrm{b}\mathrm{i}\mathrm{n}\mathrm{d}}=\:{\mathrm{G}}_{\mathrm{c}\mathrm{o}\mathrm{m}\mathrm{p}\mathrm{l}\mathrm{e}\mathrm{x}}-\:{\mathrm{G}}_{\mathrm{r}\mathrm{e}\mathrm{c}\mathrm{e}\mathrm{p}\mathrm{t}\mathrm{o}\mathrm{r}}\:-{\mathrm{G}}_{\mathrm{l}\mathrm{i}\mathrm{g}\mathrm{a}\mathrm{n}\mathrm{d}}$$

2 $$\:\varDelta\:{\mathrm{G}}_{\mathrm{b}\mathrm{i}\mathrm{n}\mathrm{d}}=\:{\mathrm{E}}_{\mathrm{g}\mathrm{a}\mathrm{s}}+{\mathrm{G}}_{\mathrm{s}\mathrm{o}\mathrm{l}}-\mathrm{T}\varDelta\:\mathrm{S}$$

3 $$\:\varDelta\:{\mathrm{E}}_{\mathrm{g}\mathrm{a}\mathrm{s}}={\mathrm{E}}_{\mathrm{i}\mathrm{n}\mathrm{t}}+{\mathrm{E}}_{\mathrm{v}\mathrm{d}\mathrm{w}}+{\mathrm{E}}_{\mathrm{e}\mathrm{l}\mathrm{e}}$$

4 $$\:{\mathrm{G}}_{\mathrm{s}\mathrm{o}\mathrm{l}}={\mathrm{G}}_{\mathrm{G}\mathrm{B}}+{\mathrm{G}}_{\mathrm{S}\mathrm{A}}$$

5 $$\:{\mathrm{G}}_{\mathrm{S}\mathrm{A}}=\gamma\:\mathrm{S}\mathrm{A}\mathrm{S}\mathrm{A}$$

where $\:{\mathrm{E}}_{\mathrm{g}\mathrm{a}\mathrm{s}}$ indicate the gas phase energy; $\:{\mathrm{E}}_{\mathrm{i}\mathrm{n}\mathrm{t}}$ is the internal energy; $\:{\mathrm{E}}_{\mathrm{e}\mathrm{l}\mathrm{e}}$ the electrostatic (Coulomb) interaction, and $\:{\mathrm{E}}_{\mathrm{v}\mathrm{d}\mathrm{w}}$ the Van der Waals forces. The $\:\varDelta\:{\mathrm{E}}_{\mathrm{g}\mathrm{a}\mathrm{s}}$ is the sum of energy contributions from electrostatic and van der Waals interactions calculated using the FF14SB force field terms of the Amber simulation package. The solvation-free energy$\:\:({\mathrm{G}}_{\mathrm{s}\mathrm{o}\mathrm{l}}$) encompasses polar ($\:{\mathrm{G}}_{\mathrm{G}\mathrm{B}}$) and non-polar ($\:{\mathrm{G}}_{\mathrm{S}\mathrm{A}}$) contributions [53] while the non-polar contribution was determined based on solvent-accessible surface area (SASA) using a water probe radius of 1.4 angstroms [54]. T represents the temperature, while S is the total solute entropy. Additionally, individual amino acid contributions to the total binding free energy were evaluated through interaction energy decomposition analysis per residue [55].

## The In Vitro Cytotoxicity Assays

### Cell Line and Cell Culture

The A549 human lung cancer cell line was obtained from Cellonex^®^ in Johannesburg, South Africa. The cells were cultured in 25 cm^2^ tissue culture flasks with 5 mL of complete culture media (CCM) containing Dulbecco’s modified Eagle’s medium (DMEM) with 4.5 g/L glucose, 10% fetal bovine serum (FBS), 1% penicillin-streptomycin antibiotics, 1% L-glutamine and 2.5% HEPES buffer. The cells were incubated at 37 °C in an incubator with 5% CO_2_ and 95% air. The culture media were changed every 2 to 3 days to maintain the cells. When the cells reached approximately 90% confluence, they were washed three times with 5 mL of PBS pH 7.4 (Sigma Aldrich, South Africa) and the remaining PBS was aspirated. The cells were detached by adding 900 µL of a solution of 0.5% trypsin and 0.2% EDTA (Thermo Fisher Scientific, Waltham, Massachusetts, USA) to the flasks and placed them in an incubator until detachment. The trypsin was removed, neutralized, and the cells were resuspended in 3mL of CCM. The resulting cell suspensions were used for subculturing to assess cellular cytotoxicity. Cell count was determined using the Trypan blue exclusion method.

### Preparation of Rutin

Rutin was purchased from Sigma Aldrich (Phytolab, PHL 89270), South Africa. 3 mg of rutin was dissolved in 5% dimethyl sulfoxide (DMSO). A sample stock of 50 µL was mixed with 950 µL of CCM to make a final stock volume of 1000 µL, which was used for the different dilutions.

### MTT Cytotoxicity Assay

The MTT assay was used to assess cell viability in A549 cells. Cells were seeded in a 96-well plate at a density of 2.0 × 10^4^ cells in 150 µL of CCM per well in triplicate and incubated overnight at 37 °C with 5% CO_2_. After removing the CCM, cells were treated with various concentrations (0-500 µM) of rutin, prepared from a stock solution of 4910 µM, to determine the optimal treatment concentration. Following 24 h of treatment, 20 µL of 5 mg/mL MTT solution and 100 µL of CCM were added to the cells and incubated for 4 hours. Subsequently, the MTT solution was aspirated and 100 µL of DMSO was added to dissolve the formazan dye. Absorbance at 570 nm (reference 690 nm) was measured using a SPECTROstar^®^ Nano microplate reader (BMG LABTECH, Ortenberg, Germany), and cell viability was calculated as a percentage relative to the control.

### Luminometric and Fluorometric Assays

The safety and effects of the treatments were evaluated by luminometrically quantifying ATP, MMP, CYP3A4 and LDH levels. The A549 cells were seeded at a density of 1 × 10^^6^ cells per flask and cultured in 25 cm^2^ cell culture flasks until they reached 80% confluence. The cells were treated with different concentrations (50, 250 and 500 µM) of rutin, with an untreated flask serving as a control and exposed to CCM only. After 24 h of treatment, the media from the treated cells were collected in 15 mL sterile centrifuge tubes for the LDH assay. The treated cells were then washed with PBS, trypsinized, and counted for the quantification of CYP3A4, ATP, and MMP levels as previously described [56].

#### CYP3A4 Activity Assay

The inhibition or induction of CYP3A4 can affect the metabolism of drugs or endogenous substances, which have potential clinical implications on therapeutic outcomes. Drug metabolism and potential interactions are crucial in pharmacological and toxicological studies, particularly for cells involved in drug metabolism [57, 58]. CYP3A4 activity was measured using the P450 Glo^®^ luminescent protocol. Cells from the control and respective treatment groups were seeded in triplicate into an opaque 96-well plate at a cell density of 2 × 10^4^ cells/well in 50 µL of PBS. A reaction mix (working solution) of P450 Glo^®^ reagent was reconstituted according to the manufacturer’s instructions and 25 µL of the reagent was added to each well. Plates were incubated in the dark for 30 min at RT, and luminescence was measured using a Modulus™ microplate reader (Turner Biosystems, Sunnyvale, California, USA) in relative light units (RLU). Results were presented as a fold change relative to the control.

#### ATP Quantification Assay

The ATP assay is utilized to evaluate the impact of treatment on intracellular ATP levels. Comparing ATP levels with other intermediate metabolites and assessing cell viability can provide insights into the pathways through which the treatment exerts its effects. ATP levels can also indicate the potential cytotoxicity of bioactive compounds, as they reflect cellular stress, injury, or cell death [59]. Cellular ATP levels are critical for metabolic processes, making the ATP assay widely applicable. The Promega Cell Titer-Glo™ assay was used to measure intracellular ATP in the treated cell suspensions. The A549 cells treated with different concentrations of rutin were seeded in a 96-well luminometry plate at a cell density of 2.0 × 10^4^ cells in 50 µL of PBS per well in triplicate. ATP working solution of 25 µL was added immediately into each well, and the plate was incubated for 30 min in the dark at room temperature. ATP levels were measured using luminescence with a Modulus™ microplate reader (Turner Biosystems, Sunnyvale, California, USA) in RLU and results were presented as a fold change relative to control.

#### The Mitochondrial Membrane Depolarization Assay

The JC-10 assay is a sensitive method used to assess mitochondrial membrane potential, cellular stress, cytotoxicity and potential apoptosis [60]. The JC-10 is a cationic lipophilic dye that accumulates in mitochondria based on the membrane potential. It is concentrated in polarized mitochondria of healthy cells with high MMP, forming J-aggregates that emit red fluorescence when excited by light at 540/590 nm. Unhealthy or dead cells have depolarized mitochondrial membranes, causing the dye to remain in a monomeric form in the cytosol and emit green fluorescence at 490–525 nm. The MMP was measured using a JC-10 fluorometric assay kit (Merck, Darmstadt, Germany). Cell suspensions from the control and treated groups were dispensed into a 96-well luminometric plate in triplicate at a density of 2 × 10^4^ cells/well in 50 µL of PBS. Then, 25 µL of JC-10 stain working solution (reconstituted as per manufacturer’s instructions) was immediately added, and the plate was incubated for 30 min in the dark at room temperature (RT). Red and green fluorescence were measured using a Modulus™ microplate reader (Turner Biosystems, Sunnyvale, California, USA) at an excitation/emission wavelength of 490/525 nm and 540/590 nm, respectively. The red/green fluorescence ratio was calculated to determine MMP, and results were expressed as a fold change relative to the control.

#### The LDH Release Assay

The LDH release assay is a useful method for assessing the cytotoxicity of potential therapeutic agents by measuring cell lysis and necrosis-induced cell death. This colorimetric assay quantifies the release of LDH from damaged cells, providing a reliable indicator of cytotoxicity and cell membrane integrity [59]. High levels of LDH in the supernatant indicate cell membrane damage and lysis. The assay measures LDH enzyme activity, which is released from the cytosol into the cell culture medium when the plasma membrane is damaged. In this study, 50 µL of the supernatant from the control and treatment samples of different concentrations of rutin were added to the wells in triplicate, followed by adding 25 µL of LDH mixture solution and incubated for 30 min in the dark at RT. A stop solution of 12.5 µL was then added to each well and LDH levels were measured using luminescence intensity at 540/590 (Ex/Em) with a Modulus™ microplate reader (Turner Biosystems, Sunnyvale, California, USA). The results were expressed as a fold change relative to the control.

### Statistical Analysis

Statistical analyses were carried out using GraphPad Prism version 5.0, GraphPad Software, San Diego, CA, USA. The concentration-response inhibition equation using non-linear regression was utilized for non-linear regression analysis to determine the 50% inhibitory concentration (IC_50_) in the MTT assay. One-way analysis of variance (ANOVA) followed by Tukey post hoc test was used for multiple variable comparisons, while an unpaired t-test with Welch’s correction was used for pair comparisons of each treatment against the control in the results obtained from ATP, MMP, LDH and CYP3A4 assays. A p-value ≤ 0.05 was considered statistically significant. Origin version 6.0 was used for the analysis and visualization of RMSD, RMSF, RoG, PCA, and the number of hydrogen bonds.

## Results and Discussion

Molecular docking and molecular dynamics (MD) simulations are commonly used computational techniques to study protein-ligand interactions and predict binding affinity based on scoring functions and various energy terms, including electrostatics, van der Waals, and solvation contributions [61, 62]. In this study, molecular docking was utilized for the initial screening to assess the binding potential of selected flavonoids to ACE2.

The molecular docking analysis identified several flavonoids with favourable binding energies. Isorhamnetin, rutin and myricetin exhibited docking scores of -9.20, -9.10 and − 9.10 kcal/mol, respectively (Table 1). The differences in scores of the five top-ranked flavonoids are minimal. These compounds, including the co-crystallized ligand (CCL) used as a reference standard, showed comparable binding affinities and no single compound can be conclusively identified as superior based solely on the docking scores. The top-ranked flavonoids demonstrated favourable interaction patterns within the ACE2 binding site, possibly involving multiple hydrogen bonding and van der Waals interactions. The CCL exhibited a lower docking score compared to most of the top-ranked flavonoids. However, docking scores are approximate and may vary depending on the scoring function and system preparation. The limitations of docking in accurately resolving small energy differences and capturing the dynamic behaviour of protein-ligand interactions required further analysis. Thus, MD simulations were carried out to have a deeper understanding of conformational stability, residue-level interactions, and binding affinity by evaluating post-simulation parameters such as RMSD, RMSF, RoG, hydrogen bonding, per residue energy decomposition and MM/GBSA free energy calculations [51, 63].

**Table 1:** The docking scores of the various flavonoids

Compounds	Docking score (kcal/mol)
Isorhamnetin	-9.20
Rutin	-9.10
Myricetin	-9.10
Quercetin	-9.00
CCL	-8.80
Isoquercetin	-8.40

### Molecular Dynamic Simulations

Molecular docking and MD simulations are often used together to gain a more comprehensive understanding of molecular interactions between molecules [64]. Molecular docking can rapidly generate multiple binding modes, making it an effective tool for virtual screening, particularly with large compound datasets. However, docking may not capture all relevant physical interactions and is limited by the accuracy of the scoring function and conformational sampling of the ligand and receptor [63]. In docking, conformational changes generated in the receptor upon ligand binding are also observed. However, MD modeled the movement and behaviour of molecules over time to provide insights into the flexibility, conformational changes, and interactions of a ligand with a receptor in the same molecular environment [63]. The MD simulations can also uncover the energetics and kinetics of binding events and provide information on the stability and dynamics of protein-ligand complexes [65].

### Root Mean Square Deviation

**Fig. 2:**
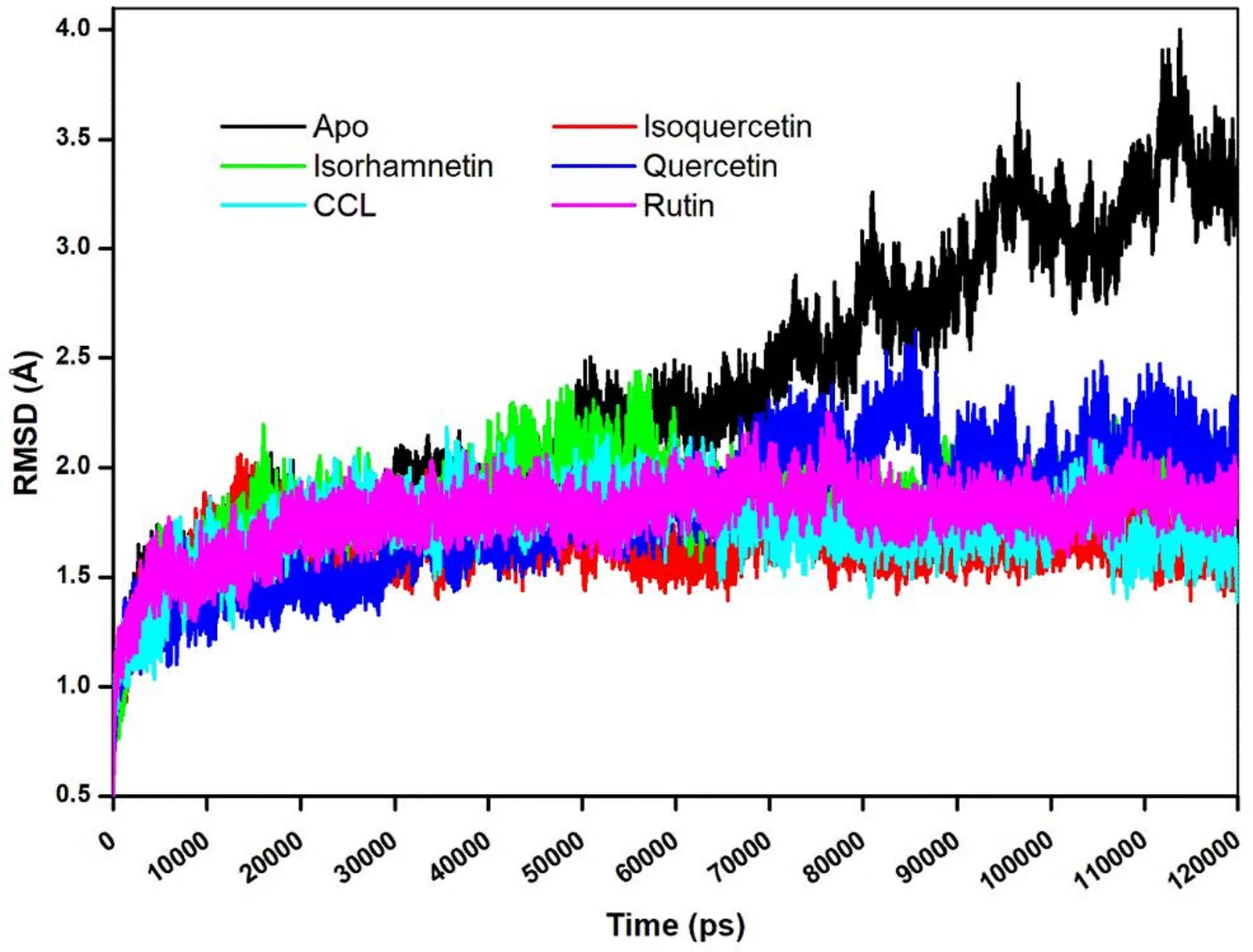
Root mean square deviation plot of isorhamnetin, isoquercetin, quercetin, rutin and CCL in complex with ACE2 and the apo during a 120 ns MD simulation

The Root Mean Square Deviation (RMSD) is a widely used metric in MD simulations to assess the structural stability of protein-ligand complexes over time, providing insights into conformational changes and similarities of biomolecules [51]. RMSD measures the average deviation of atomic positions relative to reference structures. Lower and stable RMSD values signify conformational stability and minimal deviation, while sustained increases or significant fluctuations indicate structural instability. The apo (ligand-free form of ACE2) showed a relatively high mean RMSD value of 2.40 Å, with significant deviations ranging from 2.0 to 4.0 Å after 40,000 ps (picoseconds). This indicates a lack of convergence and persistent fluctuations in the absence of ligand binding. The initial stability observed in the first 40,000 ps, followed by substantial deviations, implies that the apo structure does not maintain a stable conformational state throughout the simulation period. Conversely, the ligand-bound ACE2 complexes exhibited a clear convergence, reaching equilibrium after 10,000 ps and maintaining RMSD values consistently below 2.0 Å throughout the simulation (Fig. 2). This stable dynamic behaviour with the reduced deviation magnitude suggests conformational stability and consistent structural integrity. ACE2 complexes with rutin, isoquercetin and CCL showed lower average RMSD values of 1.78, 1.64, and 1.73 Å, respectively, indicating minimal deviation and enhanced stabilization of the receptor conformation (Fig. 2). These ligands consistently maintain RMSD values within a narrow range of less than 2.0 Å during the simulation, which indicates restricted conformational stability and strong ligand-induced stabilization within the binding pocket.

Quercetin and isorhamnetin exhibited slightly higher RMSD values of 1.81 and 1.86 Å, respectively, showing moderate structural stability but a more dynamic binding mode with conformational flexibility compared to rutin, isoquercetin and CCL. Both flavonoids reached equilibrium and remained within the acceptable range of RMSD values for stable protein-ligand complexes. The RMSD values demonstrate that ligand binding enhances ACE2 structural stability, as shown by earlier equilibration, lower deviation magnitudes and consistent plateau behaviour compared to the apo form. Notably, rutin and isoquercetin displayed the most pronounced stabilizing effects, suggesting their superior ability to maintain ACE2 conformational integrity. These results suggest these compounds could have the potential to effectively stabilize ACE2 structure and function, with possible applications in lung and cardiometabolic diseases where ACE2 is implicated. However, experimental validation and pharmacological studies are required to confirm these computational findings.

### Root Mean Square Fluctuation

Root Mean Square Fluctuation (RMSF) quantifies the flexibility of individual residues within a protein structure by measuring the average deviation of each residue from its mean position during a simulation. RMSF provides evaluation of structural stability, with lower fluctuation indicating limited residue movement and increased stability [66, 67]. It is useful in identifying flexible and stable regions of the protein, especially functionally important regions like the binding sites, which are essential for understanding ligand-induced conformational changes and potential impact on binding affinity and protein function [51, 67].

**Fig. 3:**
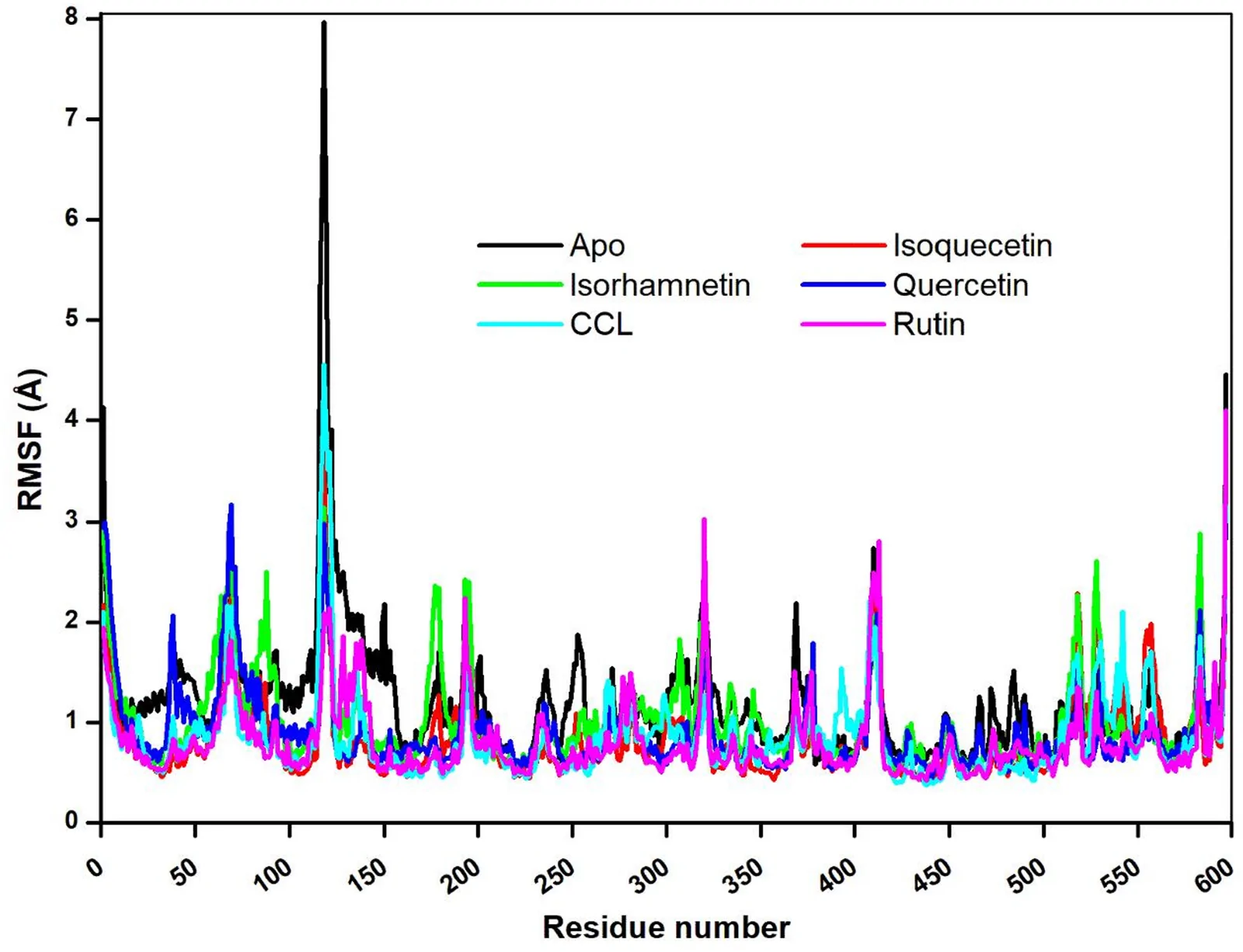
MD simulation analysis showing the root mean square fluctuation plot for isorhamnetin, isoquercetin, quercetin, rutin and CCL in complex with ACE2 and the apo during a 120 ns MD simulation

RMSF analysis showed that the Apo form of ACE2 exhibited the highest average RMSF value (1.21 Å), indicating significant residue mobility and decreased structural stability in the absence of a ligand binding (Fig. 3). This increased flexibility is consistent with its observed high and unstable RMSD (Fig. 2). This confirms that the unbound ACE2 displays a higher magnitude of residue fluctuations and conformational space. In contrast, the complexes bound to ligands showed decreased residue fluctuations, indicating that the ligands stabilize the protein structure at the residue level (Fig. 3). Interestingly, rutin exhibited the lowest RMSF (0.81 Å), followed by isoquercetin (0.84 Å) and CCL (0.85 Å), the reference ligand, indicating limited residue movement and enhanced structural stability of ACE2. The consistently low fluctuation observed suggests that ligand binding effectively constrains conformational dynamics and maintains structural integrity. Isorhamnetin displayed a relatively higher average RMSF value (1.02 Å), indicating comparatively increased residue fluctuations and a relatively less stable interaction with ACE2 compared to CCL and other flavonoid complexes. Quercetin showed moderate flexibility (0.91 Å), particularly within the N-terminal domain of ACE2, suggesting a partially dynamic binding mode. The minimal fluctuations observed for rutin and isoquercetin are consistent with their lower RMSD values (Fig. 2), reinforcing their stable binding conformations. Additionally, the low RMSF values may suggest greater selectivity and specificity, as stable binding often correlates with reduced off-target interactions. Importantly, residues within the 150–500 region, encompassing the allosteric ACE2-binding interface, remain consistently stable across ligand-bound complexes, exhibiting minimal fluctuations. The minimal fluctuation of residues within the allosteric binding site (Figs. 7 and 8, and 9) suggests persistent and stable ACE2-flavonoid interactions during the simulation. This region connects the N-terminal domain, where the receptor-binding domain (RBD) of SARS-CoV-2 attaches and the carboxylic (neck region) of ACE2 [8]. On the contrary, significant fluctuations were observed in the 100–150 residue region, particularly within the flexible loops of the N-terminal domain. These specific dynamics are likely an intrinsic flexibility rather than overall instability, which may suggest potential allosteric effects induced by stable ligand binding of distal conformational regions, potentially affecting the conformational dynamics of the receptor region that interacts with the RBD of SARS-CoV-2 [8]. The RMSF analysis demonstrates that ligand binding reduced residue flexibility within the ACE2 binding site and improved structural stability. Rutin and isoquercetin showed superior stabilizing effects and favourable binding characteristics, which support their potential as lead modulators of ACE2. Nonetheless, these findings require further investigation into structure-activity relationships and experimental evaluation to establish their therapeutic efficacy in modulating ACE2.

**Fig. 4:**
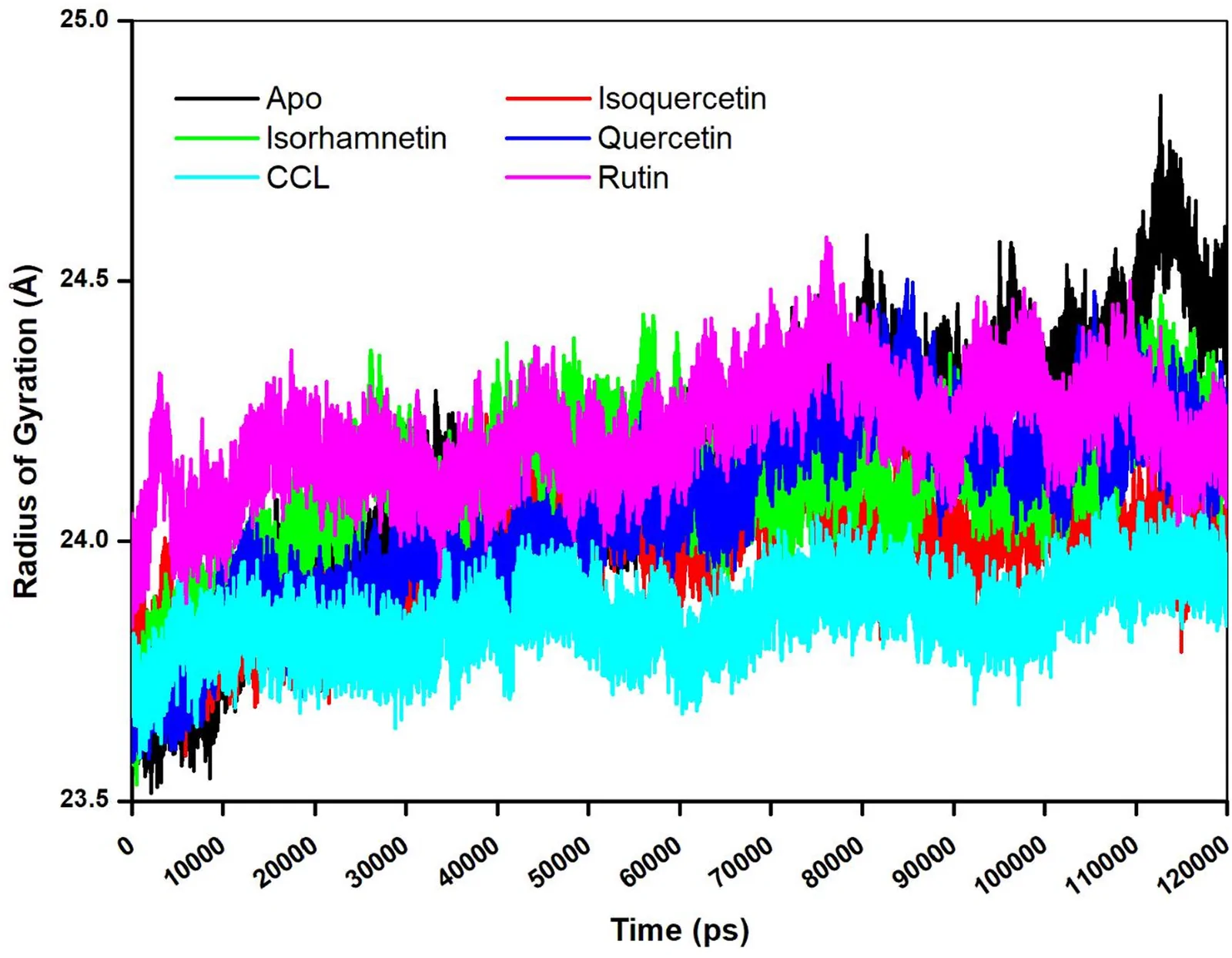
Radius of gyration plot of isorhamnetin, isoquercetin, quercetin, rutin and CCL in complex with ACE2 and the apo during a 120 ns MD simulation

### Radius of Gyration

The radius of gyration (RoG) is a fundamental parameter in molecular dynamics simulations that evaluates the compactness and structural dynamics of protein-ligand complexes. RoG assesses protein folding behavior, ligand interactions, and stability during MD simulations by measuring the degree of folding of the protein over time when bound to a ligand. This analysis tracks conformation changes in the protein throughout the simulation to provide useful insight into the stability and structural integrity of the protein-ligand complex. A significant increase in fluctuation was observed in the Apo form of ACE2, indicating instability in the protein without a ligand (Fig. 4). In the absence of a ligand, the protein lacks interactions and stability, leading to higher fluctuations compared to the ligand-bound protein. High fluctuations are often observed in a protein without a ligand during simulations [68]. Hence, comparing ligand-bound protein to its Apo is crucial for evaluating the effectiveness of a ligand in maintaining protein conformations to elicit the desired biological response.

The protein bound to the ligands remained stable after 10,000 ps of binding and maintained a relatively stable conformation until 60,000 ps, with minimal conformational changes observed during the simulation (Fig. 4). The average RoG values of the protein-ligand complexes ranged from 23.86 to 24.20 Å. Isoquercetin exhibited the most folded conformation and structural compactness after the CCL, which is the reference ligand. The CCL, with the lowest RoG, demonstrated a stable protein-ligand complex, highlighting its structural compactness optimized for the protein compared to the flavonoids. The RoG of rutin indicates its dynamic binding behavior with less rigidity in the binding modes with the ACE2 receptor. The RoG analysis showed structural compactness of the flavonoids without inducing significant conformational flexibility or protein instability during the simulation compared to the CCL. However, the minimal flexibility observed could be advantageous for targets requiring slight conformational adaptation to enhance therapeutic effects. Ultimately, the RoG values revealed that the binding of the flavonoids did not induce undesirable structural changes in the ACE2 protein that could lead to loss of function or weaker interactions.

### Hydrogen Bond Analysis

**Fig. 5:**
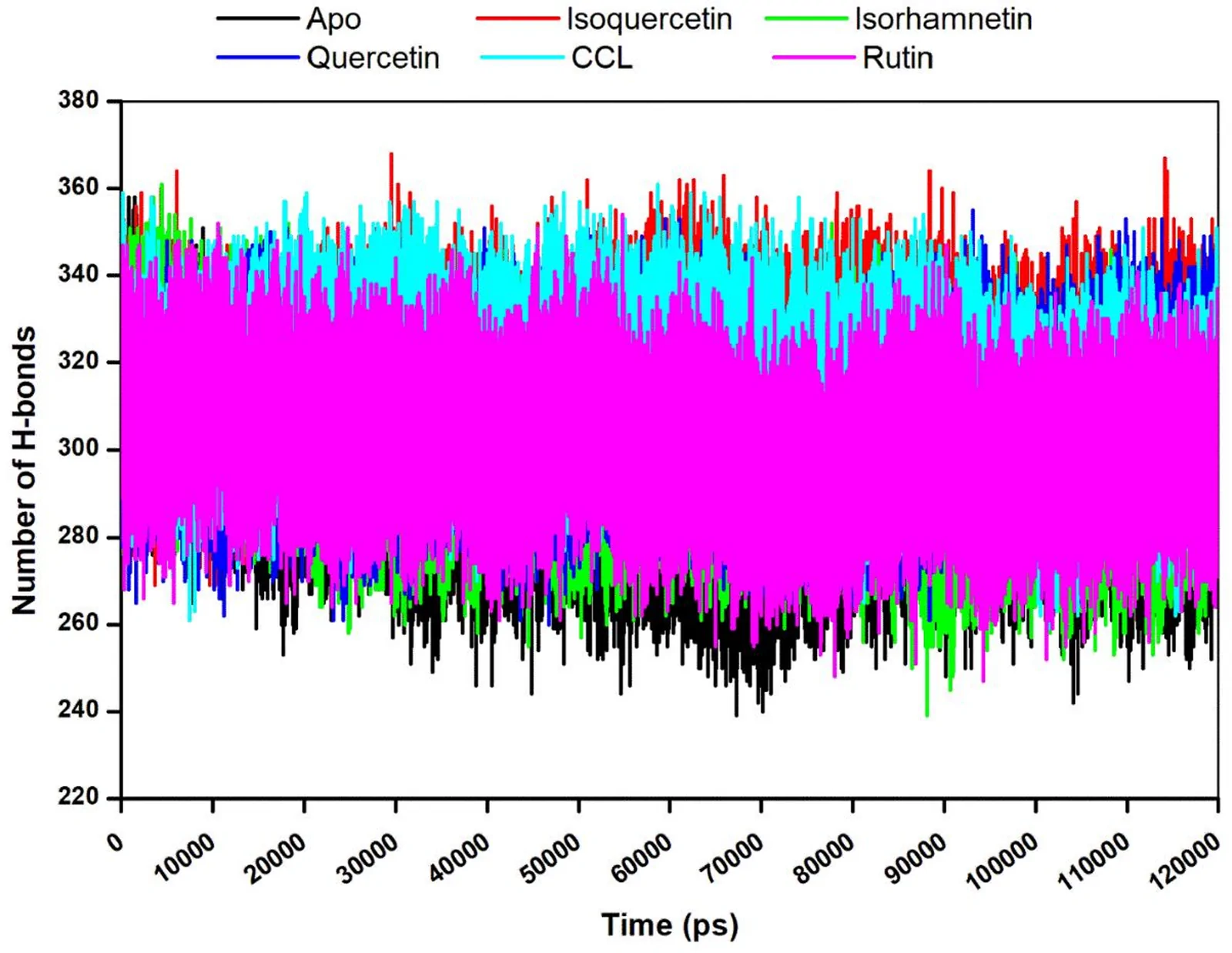
Plot showing the number of hydrogen bonding interactions for isorhamnetin, isoquercetin, quercetin, rutin and CCL in complex with ACE2 and the apo during a 120 ns MD simulation

Hydrogen bond interactions play a critical role in stabilizing protein-ligand complexes by acting as bridges between the protein and ligand [69]. These interactions mediate specific intermolecular contact with electronegative atoms like oxygen and nitrogen. The number of hydrogen bond contributions in protein-ligand complexes is significant in understanding the molecular mechanisms of recognition and binding. The number and strength of hydrogen bonds vary based on conformational changes in the protein and ligand during the binding process, influencing binding stability and affinity of the complexes [51, 69]. The total number of hydrogen bond contributions in protein-ligand interactions ranged from 293 to 314 over the course of the simulation (Fig. 5). There was a decline in hydrogen bond formations after 60,000 ps (Fig. 5). This suggests conformational adjustments within the binding pocket, which may influence ligand packing and structural rearrangements. Interestingly, isoquercetin and CCL consistently maintained higher hydrogen bond formation, indicating sustained intermolecular interactions and stability within the binding pocket. This observation aligns with their structural compactness as reinforced by the RoG analysis.

Furthermore, hydrogen bond analysis was performed to evaluate the stability of ACE2-flavonoid interactions during MD simulations. Stable hydrogen bonds were identified based on geometric criteria (donor-acceptor distance ≤ 3.5 Å) and occupancy. Residues with higher occupancy maintained stable interactions with the ligands throughout the simulation. The percentage or fraction of time that a hydrogen bond exists between the protein and ligand during the simulation is vital to the overall protein structural stability. The percentage occupancy and hydrogen bond distances of each intermolecular hydrogen bond in the ACE2-ligand complexes provide useful insights into the strength of hydrogen bonds contributing to the binding processes and structural stability of the complexes. Hence, hydrogen bond occupancy, which indicates the fraction of simulation time during which specific interactions are maintained, was analyzed to further evaluate interaction stability. High occupancy indicates persistent and stable hydrogen bonds, while lower values suggest more transient interactions. The primary residues that formed stronger hydrogen bonds in the different ACE2-ligand active sites are represented in Table 2 below. The analysis showed that VAL467 had the highest percentage of hydrogen occupancy in rutin, isoquercetin, CCL, and isorhamnetin complexes, indicating a consistently stable interaction point within the ACE2 binding site. Similarly, GLU477 exhibited high occupancy in quercetin and remained a significant interacting residue in rutin, isoquercetin, and isorhamnetin, stabilizing ligand binding. Additional residues, including ASP349, GLU384, ASP481, and ASP491, contributed to hydrogen bonding interactions, showing a network of stabilizing interactions within the binding pocket. Rutin and isoquercetin showed persistent hydrogen bonding with VAL467 and GLU477 at shorter average distances, indicating strong and stable hydrogen bonding interactions (Table 2), and contributing to interaction stability. The reference ligand CCL also showed stable hydrogen bonding with VAL467 and ASP491. The analysis of hydrogen bond distance showed that the most significant interactions occurred within a range of 2.66–2.77 Å, indicating strong hydrogen bonding interactions, which is within a good range for protein-ligand complex stability [69, 70]. The hydrogen bond analysis demonstrates that strong hydrogen bonding and high occupancy interactions were maintained throughout the simulation, contributing to the structural stability of the complexes, with rutin, isoquercetin and CLL exhibiting the most stable interactions.

**Table 2:** Hydrogen bond contributions of the varied flavonoids

	#Acceptor	Donor H	Donor	Percentage occupancy	Avg Dist
Rutin	VAL_467@O	TYR_219@HH	TYR_219@OH	97.60	2.7041
	GLU_477@OE2	ARG_159@HH22	ARG_159@NH2	97.35	2.764
	TYR_219@O	HID_223@H	HID_223@N	93.98	2.8255
	ARG_255@O	THR_431@HG1	THR_431@OG1	93.61	2.6899
	ASP_481@OD1	ARG_151@HH11	ARG_151@NH1	92.64	2.7693
**CCL**	VAL_467@O	TYR_219@HH	TYR_219@OH	98.99	2.6636
	ASP_491@OD1	TYR_165@HH	TYR_165@OH	98.69	2.6632
	GLU_384@OE1	ARG_500@HH11	ARG_500@NH1	97.58	2.7406
	GLU_477@OE2	ARG_159@HH22	ARG_159@NH2	96.95	2.7791
	ASP_481@O	SER_484@HG	SER_484@OG	94.56	2.6796
**Isoquercetin**	VAL_467@O	TYR_219@HH	TYR_219@OH	98.41	2.6689
	GLU_477@OE2	ARG_159@HH22	ARG_159@NH2	97.86	2.7577
	ASP_481@OD1	TRP_459@HE1	TRP_459@NE1	96.66	2.7565
	GLY_587@O	ARG_227@HH12	ARG_227@NH1	96.55	2.7787
	GLU_384@OE1	ARG_500@HH11	ARG_500@NH1	96.32	2.748
**Quercetin**	GLU_477@OE2	ARG_159@HH22	ARG_159@NH2	97.22	2.768
	VAL_467@O	TYR_219@HH	TYR_219@OH	97.03	2.7124
	ASP_349@O	THR_353@HG1	THR_353@OG1	95.01	2.7213
	TYR_219@O	HID_223@H	HID_223@N	94.84	2.8173
	LEU_315@O	THR_344@HG1	THR_344@OG1	94.51	2.7117
**Isorhamnetin**	VAL_467@O	TYR_219@HH	TYR_219@OH	98.27	2.6733
	GLU_477@OE2	ARG_159@HH22	ARG_159@NH2	96.94	2.7769
	TYR_219@O	HID_223@H	HID_223@N	94.33	2.8198
	ASP_207@O	THR_211@HG1	THR_211@OG1	94.25	2.7191
	THR_264@O	TYR_225@HH	TYR_225@OH	92.75	2.7126

### Molecular Mechanics/Generalized Born Surface Area Analysis

The Molecular Mechanics Generalized Born Surface Area (MM/GBSA) analysis provides an important understanding of protein-ligand interactions. This method estimates binding free energy by combining molecular mechanics and solvation energy components [71]. The binding free energy indicates the strength and attractiveness of protein-ligand interactions, with more negative values indicating stronger and more favourable binding [72]. This computational technique decomposes the total binding free energy (ΔG_bind_) into van der Waals (ΔG_vdw_), electrostatic (ΔE_ele_), and solvation (ΔG_sol_) energy contributing to ligand binding (Table 3). The most negative ΔG_bind_ values (< -30 kcal/mol) indicate stable and energetically strong binding, while greater negative ΔGbind values suggest weak and unfavourable binding interactions. Ligands with strong binding energy to their target proteins can be prioritized for further investigations as lead Compounds. Hence, the binding free energy can help in screening a large pool of ligands to identify promising ones for further experimental evaluation. This is highly significant because molecular recognition and attraction are fundamental in intracellular signal transduction for several physiological functions [73]. Additionally, the selectivity and strength of a ligand binding to a target protein are crucial for its therapeutic activity and efficacy.

**Table 3:** MMGBSA binding free energy contributions for ACE2 in complex with the respective ligands

	ΔG_vdw_	ΔE_ele_	ΔG_sol_	ΔG_bind_
Rutin	-58.71 ± 5.18	-77.87 ± 16.06	80.14 ± 9.69	-56.44 ± 8.31
CCL	-42.56 ± 4.61	230.10 ± 15.99	-234.42 ± 15.55	-46.88 ± 13.94
Isoquercetin	-47.51 ± 3.65	-69.11 ± 1594	70.09 ± 9.85	-46.53 ± 7.02
Quercetin	-31.99 ± 3.79	-48.82 ± 9.43	47.95 ± 6.09	-32.86 ± 3.41
Isorhamnetin	-32.35 ± 3.79	-13.77 ± 8.62	28.35 ± 7.39	-17.77 ± 4.07

Rutin demonstrated the most favourable binding free energy (-56.44 ± 8.31), indicating strong binding affinity compared to other ligands, followed by CCL (-46.88 ± 13.94) and then isoquercetin (-46.53 ± 7.02). Conversely, isorhamnetin (-17.77 ± 4.07) exhibited the least favourable binding free energy, while quercetin showed a relatively moderate binding energy (-32.86 ± 3.41). Moreover, rutin (-58.71 ± 5.18) and isoquercetin (-47.51 ± 3.65) showed the strongest van der Waals contributions, indicating that non-polar interactions played a significant role in stabilizing these complexes. These findings suggest that rutin and isoquercetin have a higher and enhanced binding potential towards ACE2, the target protein. In contrast, quercetin, which is mostly evaluated against SARS-CoV-2 viral proteins [74, 75], displayed weaker van der Waals interactions (-31.99 ± 3.79) consistent with its lower binding affinity compared to CCL, rutin and isoquercetin. Interestingly, rutin, reported as a potent inhibitor of the SARS-CoV-2 main protease (Mpro) [76, 77], also exhibited a highly favourable electrostatic contribution (-77.87 ± 16.06), followed by isoquercetin (-69.11 ± 1594), suggesting significant electrostatic interactions, likely due to hydrogen bonding and dipole interactions. However, the opposing effects of the corresponding polar solvation energies may counteract the electrostatic contribution, influencing electrostatic stabilization and binding free energy. Intriguingly, CCL showed a significantly high positive electrostatic energy (230.10 ± 15.99), indicating electrostatic repulsion. However, this unfavourable effect was partly compensated by its strong negative polar solvation energy (-234.42 ± 15.55), resulting in a favourable binding free energy compared to quercetin and isoharmnetin (Table 3). This shows the importance of taking into consideration the compensatory interactions between electrostatic and solvation components in understanding binding energy interactions. Isorhamnetin exhibited relatively low electrostatic contributions (-13.77 ± 8.62), indicating weaker electrostatic interactions, which contributed to its reduced binding affinity. Quercetin, a lipophilic aglycone with a basic structure of flavonoids, showed moderate binding energy, most likely influenced by a combination of its van der Waals and electrostatic interactions. However, rutin and isoquercetin, glycosylated derivatives of quercetin with a sugar moiety attached to the hydroxyl group at the C-3 carbon, have consistently shown strong binding energies influenced by both van der Waals and electrostatic contributions. These binding affinities of rutin and isoquercetin occurred from a balanced interplay of favourable intermolecular interactions and solvation effects. The sugar moieties in these compounds increase hydrophilicity, which may enhance solvation effects and promote additional intermolecular interactions within the binding site. Nonetheless, glycosylation, which increases hydrophilicity, also impacts absorption and tissue distribution and bioavailability in certain tissues. Flavonoids’ sugar moiety and the presence of hydroxyl groups have been reported to significantly contribute to strong binding affinity and stabilization in the binding site [78, 79]. Thus, this may imply prolonged actions with broader and enhanced therapeutic efficacy. However, this requires further in vivo and in vitro evaluations for confirmation. Moreover, Isorhamnetin, a methylated quercetin derivative with the hydroxyl group’s replacement at C-3’ on the B-ring by a methoxy group (-OCH3), displayed the lowest binding free energy. These suggest that glycosylation at the C-3 carbon may contribute to bioactivity by facilitating interaction with cellular targets in certain tissues. Conversely, methylation at C-3’ on the B-ring of the flavonoid backbone may suggest reduced potency, as observed with the binding free energy of isorhamnetin. Nonetheless, this may increase lipophilicity, enhancing isorhamnetin absorption and bioavailability. The binding free energy calculations indicate that non-polar, van der Waals interactions mainly influence ligand binding, with electrostatic interactions also playing a significant role that is influenced by polar solvation effects. Furthermore, the findings demonstrate that structural modifications like glycosylation in the flavonoid backbone can improve binding affinity by enhancing hydrophobic and polar interactions within the binding site.

Hence, rutin, isoquercetin, and quercetin remain promising lead compounds for consideration and further evaluation due to their favourable binding free energies and ability to form multiple molecular interactions, including hydrophobic and electrostatic interactions. However, their binding affinities are the result of a complex interplay of different energy contributions rather than a contribution from a single dominant energy interaction. These necessitate further investigations and experimental validation of these findings.

### Per Residue Energy Contribution

**Fig. 6:**
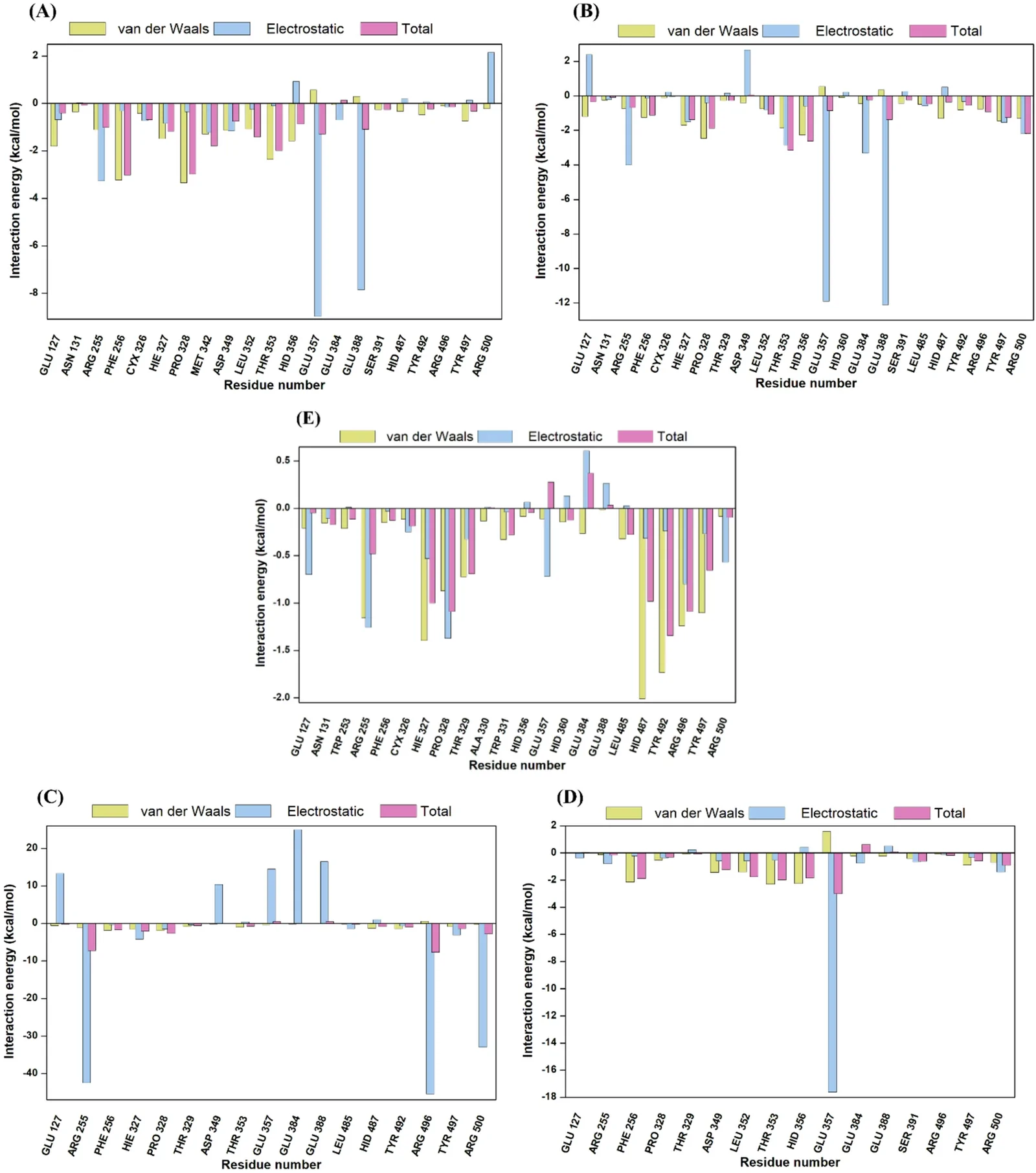
Per residue energy contribution plot: (**A**) Rutin, (**B**) Isoquercetin, (**C**) CCL, (**D**) Quercetin, (**E**) Isorhamnetin

The per residue energy contribution analysis provides impressive information on protein-ligand interactions. It allows the quantification of the contributions of different types of interactions, such as electrostatic and van der Waals, to the total binding energy [67, 80]. This computational technique helps to extract active contributions that strongly correlate with the binding affinities and specify the residues that play important roles in protein-ligand interactions. Therefore, identifying and comparing the energy decomposition of the different key residues and interactions that determine the binding specificity and selectivity in the protein-ligand complexes of the ACE2 receptor, and the flavonoids would facilitate further evaluation and optimization of the flavonoids as potential drugs. The per residue energy contributions in Fig. 6 show the different residues of the protein for the energy contributions for each of the energy interactions, van der Waals and electrostatic, to the total energy in the protein-ligand complexes of the ACE2 receptor and the flavonoids. The electrostatic energy interactions, particularly involving residues GLU357 and GLU388, dominate in rutin, isoquercetin, and quercetin, with the highest negative electrostatic energies of -8.97, -12.1, and − 17.606 kcal/mol, respectively.

Rutin and isoquercetin exhibited positive electrostatic interactions of 2.161 and 2.674 kcal/mol, respectively, but also showed negative electrostatic energy. Additionally, rutin, isoquercetin, and quercetin have substantial van der Waals contributions of -3.347, -2.467, and − 2.294 kcal/mol, respectively. Conversely, CCL with notably small van der Waals interactions exhibited a highly significant negative electrostatic energy of -45.453 kcal/mol with ARG496 and ARG255 residues forming a significant electrostatic interaction (Fig. 6). The CCL also had the highest positive electrostatic contributions of 24.96 kcal/mol with GLU357 and GLU388 as the major residues. The van der Waals energy contribution was the most significant interaction observed for isorhamnetin (-2.009 kcal/mol), although less in magnitude compared to rutin and isoquercetin. Isorhamnetin also exhibited the lowest binding free energy (Table 3), suggesting weaker hydrophobic interactions with the ACE2 binding site. This indicates a less stable binding conformation and a consequent decrease in binding affinity. Hydrophobic interactions are crucial for enhancing both binding affinity and complex stability by promoting effective contact between the ligand and protein surfaces [78, 81]. The analysis of energy values indicates that electrostatic interactions are the main energy contributors in the protein-ligand complexes of the ACE2 receptor and the ligands. These interactions are crucial for ligand binding, affinity, and stability of the protein-ligand complex, as negative electrostatic energies promote attractive forces between oppositely charged species. Similarly, van der Waals interactions also contribute to stabilizing the ligand within the binding site.

### Binding Mode Analysis and ACE2-Ligand Interactions

**Fig. 7:**
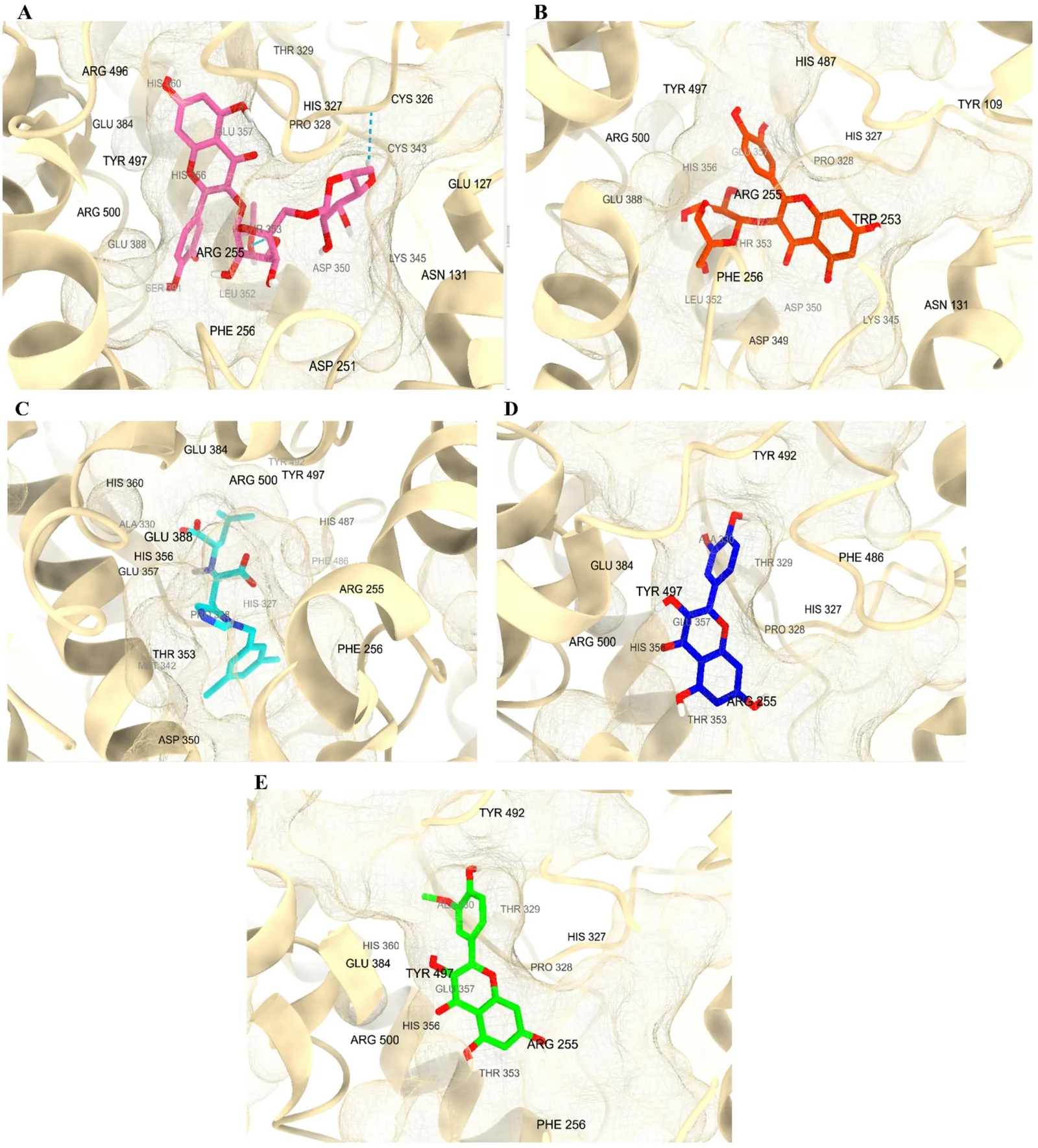
The binding modes of: (**A**) Rutin, (**B**) Isoquercetin, (**C**) CCL, (**D**) Quercetin, (**E**) Isorhamnetin at the allosteric site of ACE2

**Fig. 8:**
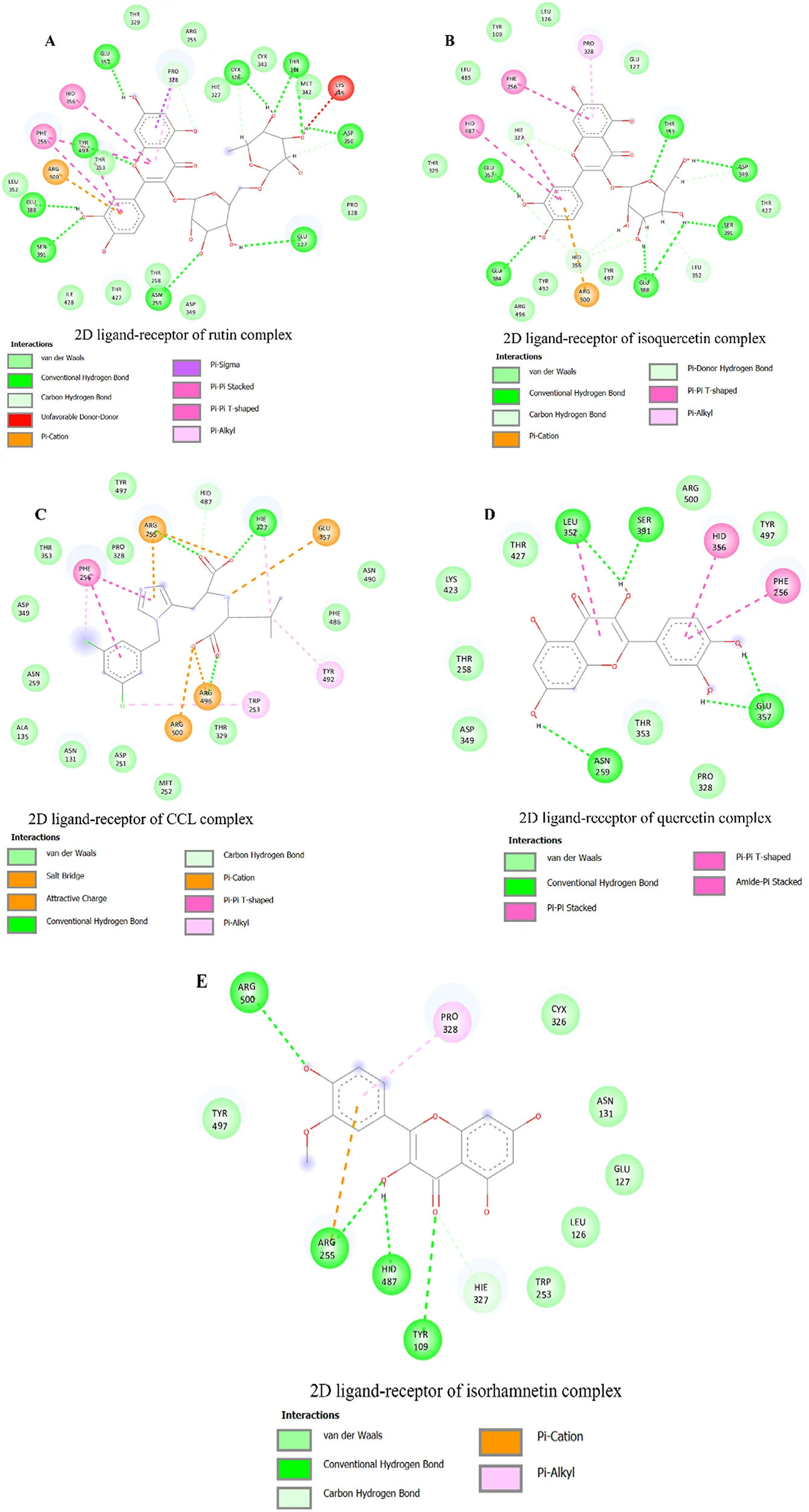
The 2D molecular interactions of: (**A**) Rutin, (**B**) Isoquercetin, (**C**) CCL, (**D**) Quercetin, (**E**) Isorhamnetin with active site residues

The ACE2 receptor protein and ligand interactions are useful in understanding the molecular mechanisms of binding and affinity based on residue-specific interactions at the ligand-binding site of the ACE2 protein. The protein-ligand complexes identified the binding modes of the compounds, and the main residues participated and contributed to the stability of the complexes (Figs. 7 and 8).

The Analysis of the interactions of the ACE2 and the flavonoids reveals hydrogen bond interactions, van der Waals, σ-π stacking interactions, and salt-bridge/charge attraction in the CCL, which contributed most to the binding of the flavonoid ligands to the ACE2 (Fig. 8). The key residues in the binding pocket of the protein include ASN259, ARG255, PHE256, PRO328, GLU357, HID356, GLU384, GLU388, SER391, HID487, TYR492, ARG496, and ARG500 (Fig. 8). However, the ACE2 interaction with the ligands was mainly hydrogen bonds of the hydroxyl groups of its phenolic ring with the GLU384 and GLU388 residues. Additionally, ARG500 showed a considerable interaction with the phenolic rings of the rutin and isoquercetin. Similarly, ARG255 and ARG500 played an important role in the interaction via salt-bridge and attractive charges with the CCL. Arginine residues (ARG255 and ARG500) often interact with negatively charged ligands or participate in salt bridges due to their positively charged guanidinium groups, as shown in Fig. 8. Moreover, the PHE256 and PRO328 residues of the ACE2 interacted directly in a water-mediated way with the phenolic rings/ribose part of the ligands. Rutin formed hydrogen bonds with GLU127, ASN259, CYX326, THR344, ASP350, GLU357, GLU388, SER391 and TYR492 residues. Rutin, like its interactions with the SARS-CoV-2 main protease M^pro^ (Agrawal et al., 2021), formed several hydrogen bonds and σ-π stacking interactions with the ACE2 receptor. Additionally, isoquercetin formed hydrogen bonds with ASP349, THR353, LEU352, HID356, GLU357, GLU384, GLU388 and SER391 residues. This demonstrates the selectivity and the binding affinity of rutin and isoquercetin to the ACE2 receptor compared to the CCL used as a reference ligand, which formed hydrogen bonds with only the HIE327 residue.

Furthermore, the Important residues interactions of the ACE2 protein in complex with the flavonoids exposed a binding pocket of the ACE2 that is distinct from its enzymatic catalytic activity (HIS374, HIS378, GLU402, LYS562, ASP368, and TYR515) and the active binding site (LYS 31, GLU35, ASP38, and LYS353) of the receptor-binding domains (RBD) of the SARS-CoV-2 [82, 83]. This signifies an allosteric binding to the ACE2 receptor by the flavonoids, representing a promising therapeutic approach to inhibit the viral binding to ACE2. Unlike orthosteric inhibitors, which bind directly to the enzymatic active site, allosteric modulators interact with distinct, non-catalytic sites. This indirect mechanism allows modulation of ACE2 to disrupt viral binding while preserving its physiological role in the RAAS. This strategy offers significant therapeutic benefits, including reduced adverse effects and a lower risk of resistance caused by viral mutations. Allosteric modulation of ACE2 shows promise in inhibiting viral entry, particularly through the highly malleable RBD of the SARS-CoV-2 spike protein, without disrupting its essential enzymatic activity [84, 85]. The development of potential allosteric inhibitors or modulators as effective therapeutics has primarily been focused on targeting SARS-CoV-2 spike proteins, particularly by stabilizing the RBD in its inactive conformation [86–88]. However, a recent study by Kalashnyk et al. (2024) found that activating α7 nicotinic acetylcholine receptors (α7nAChRs) with agonists or type II positive allosteric modulators (PAM2) inhibited the binding of SARS-CoV-2 RBD to ACE2 in a U373 cell model. This inhibition of the interaction between the SARS-CoV-2 RBD and ACE2 through activation of α7nAChRs depicts a form of allosteric modulation and a promising host-directed therapeutic approach. This disruption indicates a host-directed allosteric mechanism, where α7nAChR activation likely influenced the conformational dynamics of ACE2 and its affinity for RBD [89]. Based on the in silico findings, it is postulated that flavonoids like rutin and isoquercetin may act as effective allosteric modulators of ACE2, selectively enhancing its enzymatic functions while concurrently inhibiting viral attachment. However, experimental evaluations and evidence are required to validate and give insight into these potential roles. These findings may assist the rational optimization of existing ligands or the design of new compounds to modulate ACE2, ultimately contributing to therapeutic advances in both infectious and cardiometabolic diseases.

### Principal Component Analysis

**Fig. 9:**
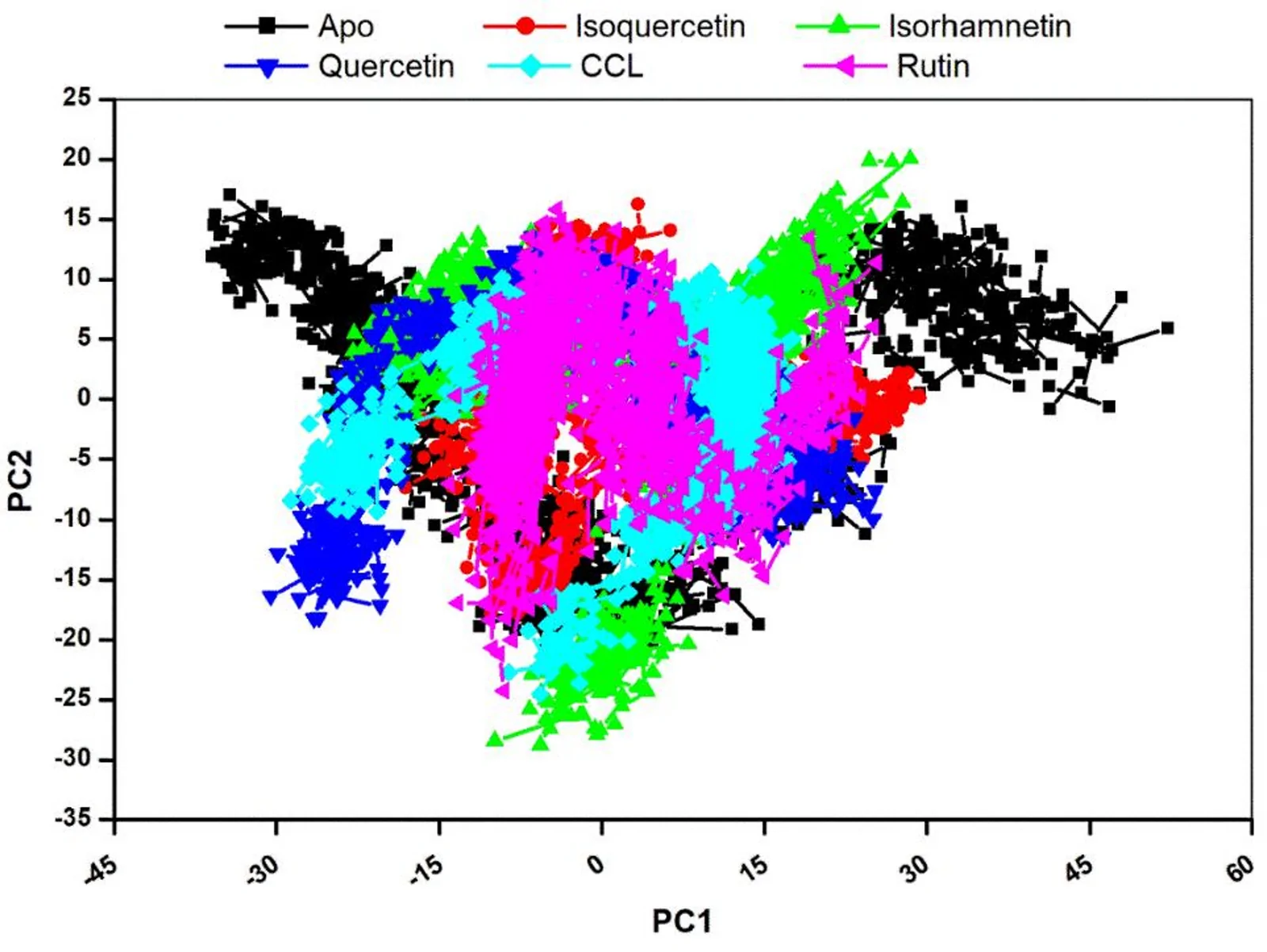
Principal component analysis (PCA) plot flavonoids (isorhamnetin, isoquercetin, quercetin and rutin), CCL in complex with ACE2 and the apo during a 120 ns MD simulation

Principal Component Analysis (PCA) is commonly used in molecular dynamics (MD) simulations to analyze the conformational changes of protein-ligand complexes over time [90]. This helps in understanding the key atomic motions that influence binding activity and identifying the major molecular motions contributing to ligand binding, protein folding, and stability, which are essential for evaluating the efficacy and potential of lead compounds [90, 91]. Hence, PCA is very useful in assessing the binding impact of ligands on the structural stability and conformational flexibility of the target protein. The PCA plot in Fig. 9 displayed compactness, conformational changes, and variations in the protein-ligand complexes during the molecular dynamics simulation. Rutin and isoquercetin clustered closely around the mean with minimal deviations from zero (Fig. 9), indicating balanced and stable interactions with the target protein without significant conformational changes. This aligns with the findings from the RMSD, MM/GBSA, and RoG analyses, which suggest good stability and effective binding of rutin and isoquercetin with the target protein without causing structural destabilization. Conversely, the Apo structure showed the highest variability and spread, reflecting significant fluctuation and structural instability of the protein in the absence of a ligand.

### Cytotoxicity and Metabolic and Mitochondrial Effects of Rutin on A549 Cells

The use of computational methods and bioassays has continued to evolve and advance as complementary research tools for developing new therapeutics. Computational techniques, including molecular docking and MD simulations, were utilized to evaluate the binding affinities and interactions between ACE2, the target protein, and the ligands (flavonoids and CCL, the reference ligand). These methods helped to understand the contributions of each interaction to the overall binding affinity, stability and dynamic behaviour of the protein-ligand complexes [63]. Based on the in silico analysis, rutin showed the most favourable binding free energy and interactions with ACE2 compared to the other flavonoids and was prioritized for experimental evaluation. Although isoquercetin exhibited a slightly more stable complex based on its RMSD and ROG values. However, rutin has been extensively studied with significant data on its pharmacokinetics and pharmacological effects from both in vitro and in vivo studies, enhancing its translational relevance compared to isoquercetin. Nevertheless, isoquercetin is still a promising option for future experimental investigations to strengthen further comparative analysis. Cytotoxicity assays were carried out to investigate potential adverse effects and mechanisms of cellular toxicity and cell death. In vitro cytotoxicity and biochemical assays were conducted to provide insights into the safety of rutin, focusing on cell membrane integrity, mitochondrial function and cellular metabolism. The MTT (3-[4,5-dimethylthiazol-2-yl]-2,5-diphenyl tetrazolium bromide) assay and the luminometric cytotoxicity assays were used to evaluate the effects of rutin on ATP levels, mitochondrial membrane stability, lactate dehydrogenase (LDH) release, and CYP3A4 enzyme activity in A549 cells, providing insights into cell membrane integrity and cellular energy metabolism, respectively, with cell viability. These assays play a crucial role in screening potential therapeutic agents for safety [59, 92, 93].

#### MTT Assay

**Fig. 10:**
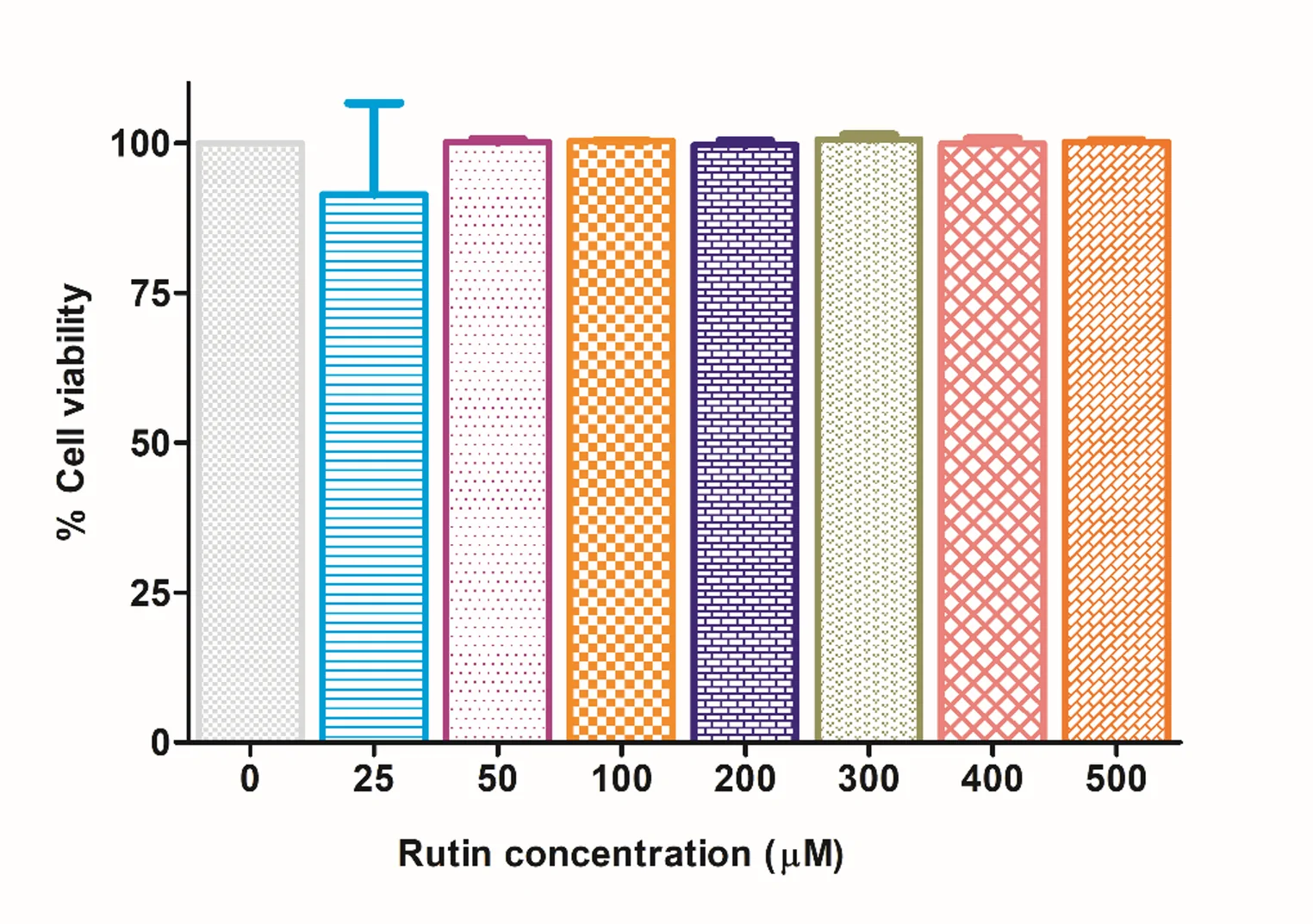
Percentage cell viability of the different concentrations of rutin (25–500 µM) on A549 cells after 24 h. There was no cytotoxicity observed in the MTT assay used to determine the cell viability of A549. Results were analyzed using a one-way ANOVA test followed by the Tukey post hoc test to compare means from *n* = 3 technical replicates. *p ˂ 0.05 is considered statistically significant

The MTT assay measures cell viability by assessing mitochondrial activity, which reflects cell metabolic activity and viability. In this colorimetric assay, the MTT (yellow tetrazole) is reduced to purple formazan by metabolically active cells [92]. The cell viability at the lowest concentration in the treatment groups (Fig. 10) showed a slight decrease to 91.4 ± 8.81%. However, this decrease is not very significant to suggest strong cytotoxic effects on the A549 cells. Based on ISO standards for in vitro cytotoxicity assessments to ensure the safety of bioactive compounds and the biocompatibility of medical devices, the percentage viability of rutin on A549 cells suggests a negligible risk of cytotoxic potential [94, 95].

The MTT cytotoxicity assay results showed that rutin did not significantly affect the viability of A549 cells at higher concentrations, where cell viability remained around 100% (Fig. 10). This indicates that A549 cells maintained their metabolic activity and cellular health within the tested concentrations, aligning with the reported in silico toxicity prediction of rutin [77]. Previous report by Hoai et al. (2020) using the MTT assay showed that pure rutin was minimally toxic to A549 lung cancer cells after 72 h of treatment, with 4.65% and 4.4% cell death at 150 µM and 300 µM treatments, respectively [96]. The slight decrease in viability at the lowest concentrations may indicate an adaptive cellular response or mild cytotoxic effects.

**Fig. 11:**
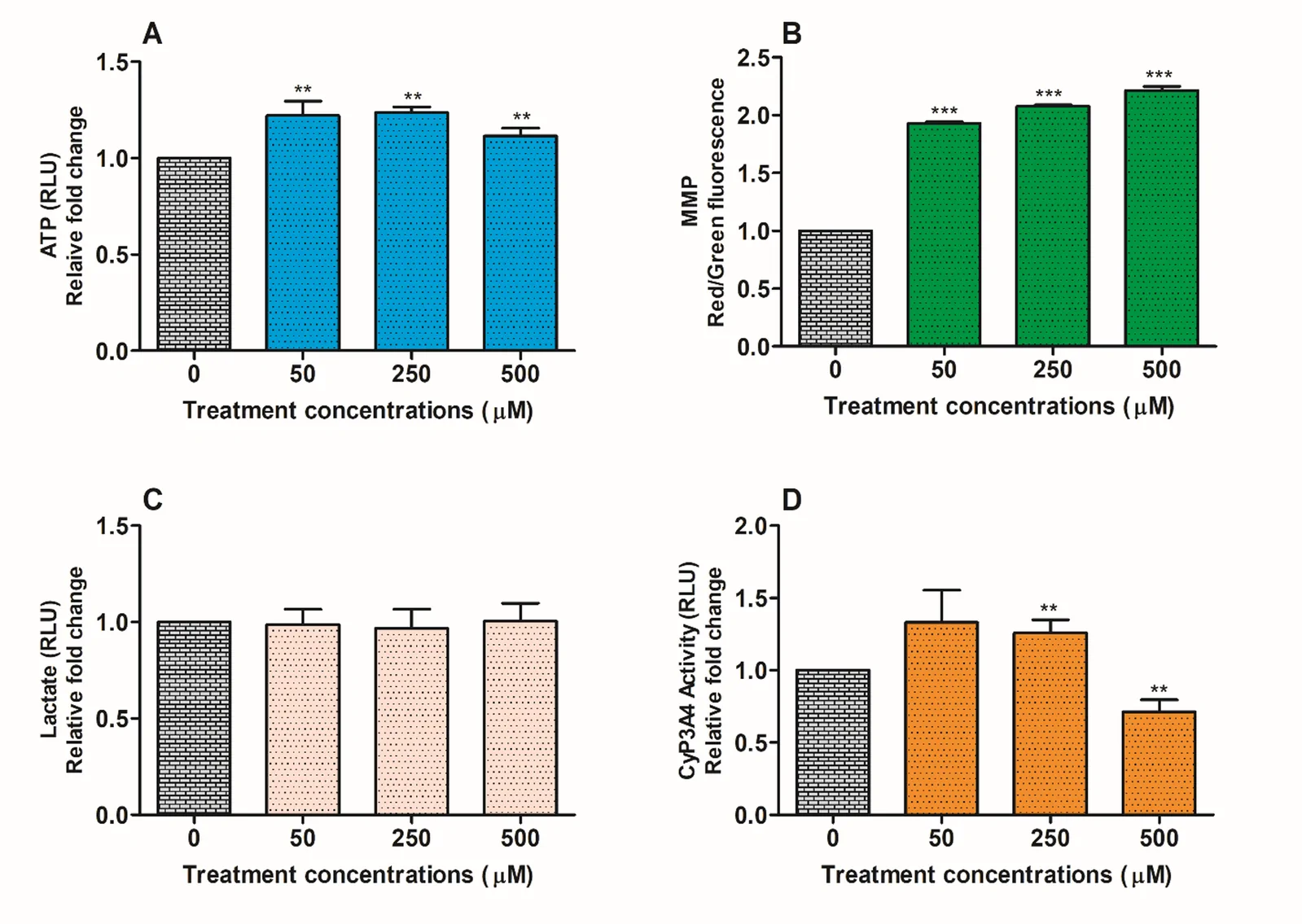
Cytotoxicity-based assays show the effects of the different concentrations of rutin (50, 250 and 500 µM) on the A549 cells after 24 h of treatment on: (**A**) ATP activity, (**B**) mitochondrial membrane polarization (MMP), (**C**) LDH release activity, (**D**) CYP3A4 activity. Results were analyzed using a one-way ANOVA test followed by the Tukey post hoc test to compare means. The results are presented as mean ± SD, (relative fold change) from *n* = 3 technical replicates for each of the biological replicates (*n* = 4) *p ˂ 0.05 is considered statistically significant (*p < 0.05, **p < 0.01, ***p < 0.001)

The MTT assay results show that cell viability remained relatively unchanged, indicating that the tetrazolium salt was converted to formazan only in viable cells with intact mitochondrial function [93, 97]. This demonstrates that rutin did not adversely affect mitochondrial integrity. Mitochondria play a crucial role in ATP production, which supports various cellular processes. Therefore, assessing cellular ATP levels is important for evaluating the effects of treatments on cell viability and cellular energy dynamics. The increased ATP levels observed at all concentrations of rutin (Fig. 11A) suggest that mitochondrial function was not compromised, allowing cells to remain viable (Fig. 10). Additionally, the increase in ATP levels indicates improved cellular energy status that may be associated with the modulation of signaling pathways involved in ATP synthesis or utilization. ATP levels are linked to several metabolic processes and a variety of mechanisms. Hence, the observed increase alone does not differentiate between improved mitochondrial function and metabolic adaptation to cellular stress. ATP production is closely linked to mitochondrial health, and assessing mitochondrial membrane potential (MMP) provides insights into oxidative phosphorylation and cellular redox status. Rutin treatment led to a dose-dependent increase in MMP in A549 cells (Fig. 11B), indicating healthy mitochondrial function. The polarized mitochondrial membrane is characteristic of healthy cells and is essential for cell survival. However, depolarization of the mitochondrial membrane initiates cell death pathways, including apoptosis [60]. The data suggest that the rutin-treated cells maintained sufficient MMP to support ATP production and resist cellular stress, promoting survival and viability. The MMP data may also suggest minimal levels of reactive oxygen species that can disrupt membrane integrity. Overall, the MMP and ATP results indicate membrane stability and functional integrity in rutin-treated cells. However, additional studies will be needed to assess the effects of rutin on mitochondrial modulation and cellular energy pathways.

The release of LDH from damaged cells into the culture medium indicates damage to the plasma membrane. In this study, there was a minimal difference in LDH levels in A549 cells treated with rutin compared to the control (Fig. 11C), indicating that rutin did not adversely affect membrane integrity. The data suggests minimal stress without enhancing cellular damage. LDH is crucial for anaerobic glycolysis, converting pyruvate to lactate [98]. In the process, NADH is oxidized to NAD^+^, and the electrons are channeled to the glycolytic pathway to create ATP [99]. This aligns with the increased ATP levels in this study (Fig. 11A). Decreased LDH activity has been linked to improved insulin sensitivity and glucose homeostasis [100, 101], which indicates potential therapeutic benefits of rutin in diabetes. The significant increase in ATP and MMP, along with stable LDH levels in the A549 cells, shows that rutin may have broader therapeutic benefits in lung diseases and protective effects on mitochondrial function by modulating mitochondrial biogenetic pathways [99]. Compounds with protective effects, such as anti-inflammatory and antioxidant properties, or cytoprotective effects, can help cells resist stress and prevent cell damage, thereby enhancing cell survival [102, 103].

Cytochrome P450 3A4 (CYP3A4) is an important metabolic enzyme expressed in the lungs, with higher levels predominantly in the liver and small intestine. It plays a vital role in metabolizing endogenous substrates, xenobiotics and various therapeutic drugs [58, 104]. Assessing the effect of lead compounds on CYP3A4 activity is important for understanding potential drug interactions and the pharmacokinetics of concomitantly used medication in terms of bioavailability and safety [105, 106]. Rutin was found to enhance CYP3A4 activity at lower concentrations (50 and 250 µM) but inhibit it at higher concentrations (500 µM), as shown in Fig. 11D. This dual effect of rutin suggests a concentration-dependent modulation of CYP3A4, which may have implications for drug metabolism, therapeutic efficacy and safety. The increase in CYP3A4 activity at lower rutin doses may improve drug clearance, potentially reducing toxicity and enhancing cell survival [107]. Conversely, inhibition at higher doses could limit drug metabolism, which necessitates caution when co-administering with other therapeutic agents. The ability of rutin to selectively regulate CYP3A4, as observed in this study, may provide therapeutic advantages by balancing efficacy and safety without significant clinical consequences [107, 108]. A study by Alyami et al. (2023) reported the inhibitory effects of rutin in hepatic and pancreatic cells [109], signifying that its impact may vary in different tissues. However, further studies are required to validate the consistency of these effects in other cellular in vitro models.

## Conclusions

This study investigated the interactions between selected flavonoids and the ACE2, an essential protein implicated in the pathophysiology of COVID-19 and cardiometabolic diseases. Using computational methods, the findings provided structural insights into ligand binding, conformational stability and interaction patterns of the flavonoids with ACE2, which could be relevant for ACE2-target drug design and its potential modulation. Molecular docking and MD simulations showed that rutin and isoquercetin formed stable complexes with ACE2, supported by favourable binding energies and consistent trajectory metrics, including RMSD, RMSF, RoG and MM/GBSA analyses. Both compounds interacted with key residues ARG255, PRO328, GLU357, GLU384, GLU388, and ARG500 in the binding pocket, suggesting a potential allosteric binding mechanism. The observed binding did not involve disruption of the catalytic domain, indicating that ACE2 enzymatic function may be preserved under such conditions. Complementary In vitro assays demonstrated that rutin was non-cytotoxic to A549 cells at the tested concentrations and was associated with improved cellular energy status, indicating a favourable safety and metabolic profile. This is important for potential lead compounds, where cellular safety and metabolic stability are key factors for further development. These findings provided a preliminary complementary in vitro study to the computational findings, but do not directly establish ACE2 modulation or antiviral activity. The results suggest that these flavonoids, particularly rutin, could be a potential lead compound for targeting ACE2 through non-catalytic interactions. However, the promising modulatory potential and therapeutic implications are mainly based on computational predictions and preliminary in vitro results. Pharmacological studies are needed to confirm the ability of these compounds to influence ACE2 function or interfere with SARS-CoV-2 binding. While molecular docking and dynamics simulations indicate stable interactions between rutin and ACE2. There are limitations in this study, including a lack of direct experimental studies to confirm ACE2 modulation through enzymatic activity assays, biophysical binding measurements, and protein or gene expression analysis to verify the predicted interactions. The study also did not include viral entry or spike-ACE2 binding assays, so the relevance of these findings to viral entry mechanisms remains to be determined. Furthermore, A549 cells, widely used as a lung epithelial model, have low endogenous ACE2 expression, which may limit their suitability in functional investigations involving ACE2 compared to certain primary airway epithelial systems. Future studies should consider using ACE2-overexpressing cell models, primary airway epithelial cells, viral entry assays and targeted mechanistic studies to verify the predicted interactions of ACE2-flavonoid complexes and elucidate the biological significance of rutin and isoquercetin on ACE2 signalling and interference with SARS-CoV-2 binding.

Hence, this study focused on computational analyses for understanding certain flavonoid interactions with ACE2, identifying rutin as a compound with stable interactions with the ACE2 receptor and is non-cytotoxic with beneficial effects on cellular metabolic activity in A549 cells. These results contribute to the exploration of natural compounds that may interact and serve as potential modulators of host proteins involved in important physiological and disease-related pathways. Finally, these computational findings provided a foundation for future mechanistic studies and suggested a potential structural basis for interference with SARS-CoV-2 binding to the ACE2 receptor. However, do not constitute direct evidence of ACE2 modulation or antiviral activity.

## Data Availability

The data that support the findings of this study are available from the corresponding author upon reasonable request.

## References

[1] Omrani, M., Keshavarz, M., Nejad Ebrahimi, S., Mehrabi, M., McGaw, L. J., Ali Abdalla, M., & Mehrbod, P. (2021). Potential Natural Products Against Respiratory Viruses: A Perspective to Develop Anti-COVID-19 Medicines. *Frontiers in Pharmacology*, *11*. 10.3389/fphar.2020.586993 PMC7926205 33679384

[2] Haddad, M., Gaudreault, R., Sasseville, G., Nguyen, P. T., Wiebe, H., Van De Ven, T., Bourgault, S., Mousseau, N., & Ramassamy, C. (2022). Molecular Interactions of Tannic Acid with Proteins Associated with SARS-CoV-2 Infectivity. *International Journal Of Molecular Sciences*, *23*(5). 10.3390/ijms23052643 PMC8910432 35269785

[3] Satarker, S., & Nampoothiri, M. (2020). Structural Proteins in Severe Acute Respiratory Syndrome Coronavirus-2. *Archives of Medical Research*, *51*(6), 482–491. 10.1016/j.arcmed.2020.05.012 32493627 PMC7247499

[4] Toigo, L., Dos Santos Teodoro, E. I., Guidi, A. C., Gancedo, N. C., Petruco, M. V., Melo, E. B., Tonin, F. S., Fernandez-Llimos, F., Chierrito, D., De Mello, J. C. P., De Medeiros, D. C., Araújo, A. C. C., & Sanches (2023). Flavonoid as possible therapeutic targets against COVID-19: a scoping review of in silico studies. *DARU Journal of Pharmaceutical Sciences*, *31*(1), 51–68. 10.1007/s40199-023-00461-3 37195402 PMC10191091

[5] Sender, R., Bar-On, Y. M., Gleizer, S., Bernshtein, B., Flamholz, A., Phillips, R., & Milo, R. (2021). The total number and mass of SARS-CoV-2 virions. *Pnas* 118(25), e2024815118 . 10.1073/pnas.2024815118 PMC8237675 34083352

[6] Piccoli, L., Park, Y. J., Tortorici, M. A., Czudnochowski, N., Walls, A. C., Beltramello, M., Silacci-Fregni, C., Pinto, D., Rosen, L. E., & Bowen, J. E. (2020). Mapping neutralizing and immunodominant sites on the SARS-CoV-2 spike receptor-binding domain by structure-guided high-resolution serology. *Cell*, *183*(4), 1024–1042. 32991844 10.1016/j.cell.2020.09.037 PMC7494283

[7] Yarmohammadi, A., Yarmohammadi, M., Fakhri, S., & Khan, H. (2021). Targeting pivotal inflammatory pathways in COVID-19: A mechanistic review. *European Journal of Pharmacology*, *890*, 173620. 10.1016/j.ejphar.2020.173620 33038418 PMC7539138

[8] Lubbe, L., Gyles, D., Oosthuizen, K., & Edward (2020). ACE2 and ACE: structure-based insights into mechanism, regulation and receptor recognition by SARS-CoV. *Clinical Science*, *134*(21), 2851–2871. 10.1042/cs20200899 33146371 PMC7642307

[9] Ingraham, N. E., Barakat, A. G., Reilkoff, R., Bezdicek, T., Schacker, T., Chipman, J. G., Tignanelli, C. J., & Puskarich, M. A. (2020). Understanding the renin–angiotensin–aldosterone–SARS-CoV axis: a comprehensive review. *European Respiratory Journal*, *56*(1), 2000912. 10.1183/13993003.00912-2020 32341103 PMC7236830

[10] Cheng, H., Wang, Y., & Wang, G. Q. (2020). Organ-protective effect of angiotensin‐converting enzyme 2 and its effect on the prognosis of COVID‐19. *Journal of medical virology*, *92*(7), 726–730. 32221983 10.1002/jmv.25785 PMC7317908

[11] Turner, A. J., & Nalivaeva, N. N. (2022). Angiotensin-converting enzyme 2 (ACE2): Two decades of revelations and re-evaluation. *Peptides*, *151*, 170766. 10.1016/j.peptides.2022.170766 35151768 PMC8830188

[12] Keni, R., Alexander, A., Nayak, P. G., Mudgal, J., & Nandakumar, K. (2020). COVID-19: emergence, spread, possible treatments, and global burden. *Frontiers in public health*, *8*, 216. 32574299 10.3389/fpubh.2020.00216 PMC7270802

[13] Stein, R. A. (2020). The 2019 coronavirus: Learning curves, lessons, and the weakest link. *International Journal of Clinical Practice* 74(4), e13488. 10.1111/ijcp.13488 PMC7165502 32052918

[14] Wang, B., Kovalchuk, A., Li, D., Rodriguez-Juarez, R., Ilnytskyy, Y., Kovalchuk, I., & Kovalchuk, O. (2020). In search of preventive strategies: novel high-CBD Cannabis sativa extracts modulate ACE2 expression in COVID-19 gateway tissues. *Aging (Albany NY)*, *12*(22), 22425–22444. 10.18632/aging.202225 33221759 PMC7746344

[15] Groß, S., Jahn, C., Cushman, S., Bär, C., & Thum, T. (2020). SARS-CoV-2 receptor ACE2-dependent implications on the cardiovascular system: From basic science to clinical implications. *Journal of Molecular and Cellular Cardiology*, *144*, 47–53. 10.1016/j.yjmcc.2020.04.031 32360703 PMC7191280

[16] Li, S., Jiang, L., Li, X., Lin, F., Wang, Y., Li, B., Jiang, T., An, W., Liu, S., Liu, H., Xu, P., Zhao, L., Zhang, L., Mu, J., Wang, H., Kang, J., Li, Y., Huang, L., Zhu, C., Zhao, S., Lu, J., Ji, J., & Zhao, J. (2020). Clinical and pathological investigation of patients with severe COVID-19. *JCI Insight*, *5*(12). 10.1172/jci.insight.138070 PMC7406259 32427582

[17] Cárdenas, G., Chávez-Canales, M., Espinosa, A. M., Jordán-Ríos, A., Malagon, D. A., Murillo, M. F. M., Araujo, L. V. T., Campos, R. L. B., Wong-Chew, R. M., González, L. E. R., et al. (2022). Intranasal dexamethasone: a new clinical trial for the control of inflammation and neuroinflammation in COVID-19 patients. *Trials*, *23*(1), 148. 10.1186/s13063-022-06075-5 35164840 PMC8845269

[18] Baker, J. R., Mahdi, M., NicolauJr., D. V., Ramakrishnan, S., Barnes, P. J., Simpson, J. L., Cass, S. P., Russell, R. E. K., Donnelly, L. E., & Bafadhel, M. (2022). Early Th2 inflammation in the upper respiratory mucosa as a predictor of severe COVID-19 and modulation by early treatment with inhaled corticosteroids: a mechanistic analysis. *Lancet Respir Med*, *10*(6), 545–556. 10.1016/s2213-2600(22)00002-9 35397798 PMC8989397

[19] Shenoy, V., Ferreira, A. J., Qi, Y., Fraga-Silva, R. A., Díez-Freire, C., Dooies, A., Jun, J. Y., Sriramula, S., Mariappan, N., Pourang, D., Venugopal, C. S., Francis, J., Reudelhuber, T., Santos, R. A., Patel, J. M., Raizada, M. K., & Katovich, M. J. (2010). The angiotensin-converting enzyme 2/angiogenesis-(1–7)/Mas axis confers cardiopulmonary protection against lung fibrosis and pulmonary hypertension. *American Journal Of Respiratory And Critical Care Medicine*, *182*(8), 1065–1072. 10.1164/rccm.200912-1840OC 20581171 PMC2970847

[20] Ciechanowicz, A. K., Lay, W. X., Prado Paulino, J., Suchocki, E., Leszczak, S., Leszczak, C., & Kucia, M. (2022). Angiotensin 1–7 Stimulates Proliferation of Lung Bronchoalveolar Progenitors—Implications for SARS-CoV-2 Infection. *Cells*, *11*(13), 2102. 35805187 10.3390/cells11132102 PMC9266020

[21] Hassler, L., Wysocki, J., Gelarden, I., Sharma, I., Tomatsidou, A., Ye, M., Gula, H., Nicoleascu, V., Randall, G., Pshenychnyi, S., Khurram, N., Kanwar, Y., Missiakas, D., Henkin, J., Yeldandi, A., & Batlle, D. (2022). A Novel Soluble ACE2 Protein Provides Lung and Kidney Protection in Mice Susceptible to Lethal SARS-CoV-2 Infection. *Journal Of The American Society Of Nephrology*, *33*(7), 1293–1307. 10.1681/asn.2021091209 35236774 PMC9257820

[22] Zhang, J., Dong, J., Martin, M., He, M., Gongol, B., Marin, T. L., Chen, L., Shi, X., Yin, Y., Shang, F., Wu, Y., Huang, H. Y., Zhang, J., Zhang, Y., Kang, J., Moya, E. A., Huang, H. D., Powell, F. L., Chen, Z., Thistlethwaite, P. A., Yuan, Z. Y., & John, Y. J. (2018). AMP-activated protein kinase phosphorylation of angiotensin-converting enzyme 2 in endothelium mitigates pulmonary hypertension. *American Journal Of Respiratory And Critical Care Medicine*, *198*(4), 509–520. 10.1164/rccm.201712-2570OC 29570986 PMC6118028

[23] Marhl, M., Grubelnik, V., Magdič, M., & Markovič, R. (2020). Diabetes and metabolic syndrome as risk factors for COVID-19, Diabetes and Metabolic Syndrome. *Clinical Research and Reviews*, *14*(4), 671–677. 10.1016/j.dsx.2020.05.013 PMC7205616 32438331

[24] Nigro, E., Perrotta, F., Polito, R., D’Agnano, V., Scialò, F., Bianco, A., & Daniele, A. (2020). Metabolic Perturbations and Severe COVID-19 Disease: Implication of Molecular Pathways. *Int J Endocrinol*, *2020*, 8896536. 10.1155/2020/8896536 33312199 PMC7703458

[25] Wang, B., Li, R., Lu, Z., & Huang, Y. (2020). Does comorbidity increase the risk of patients with COVID-19: evidence from meta-analysis. *Aging (albany NY)*, *12*(7), 6049. 32267833 10.18632/aging.103000 PMC7185114

[26] Sharma, P., Behl, T., Sharma, N., Singh, S., Grewal, A. S., Albarrati, A., Albratty, M., Meraya, A. M., & Bungau, S. (2022). COVID-19 and diabetes: Association intensify risk factors for morbidity and mortality. *Biomedicine & Pharmacotherapy*, *151*, 113089. 10.1016/j.biopha.2022.113089 35569351 PMC9080053

[27] Liu, Y., Yang, Y., Zhang, C., Huang, F., Wang, F., Yuan, J., Wang, Z., Li, J., Li, J., & Feng, C. (2020). Clinical and biochemical indexes from 2019-nCoV infected patients linked to viral loads and lung injury. *Science China Life Sciences*, *63*, 364–374. 32048163 10.1007/s11427-020-1643-8 PMC7088566

[28] Viurcos-Sanabria, R., & Escobedo, G. (2021). Immunometabolic bases of type 2 diabetes in the severity of COVID-19. *World J Diabetes*, *12*(7), 1026–1041. 10.4239/wjd.v12.i7.1026 34326952 PMC8311488

[29] Ali Abdalla, Y. O., Subramaniam, B., Nyamathulla, S., Shamsuddin, N., Arshad, N. M., Mun, K. S., Awang, K., & Nagoor, N. H. (2022). Natural products for cancer therapy: a review of their mechanism of actions and toxicity in the past decade. *Journal of Tropical Medicine*, *2022*(1), 5794350. 35309872 10.1155/2022/5794350 PMC8933079

[30] Wang, T., Li, Q., & Bi, K. (2018). Bioactive flavonoids in medicinal plants: Structure, activity and biological fate. *Asian Journal of Pharmaceutical Sciences*, *13*(1), 12–23. 10.1016/j.ajps.2017.08.004 32104374 PMC7032191

[31] Hao, B., Yang, Z., Liu, H., Liu, Y., & Wang, S. (2024). Advances in flavonoid research: Sources, biological activities, and developmental prospectives. *Current Issues in Molecular Biology*, *46*(4), 2884–2925. 38666911 10.3390/cimb46040181 PMC11049524

[32] Kumar, S., Paul, P., Yadav, P., Kaul, R., Maitra, S. S., Jha, S. K., & Chaari, A. (2022). A multi-targeted approach to identify potential flavonoids against three targets in the SARS-CoV-2 life cycle. *Computers in Biology and Medicine*, *142*. 10.1016/j.compbiomed.2022.105231 PMC8750703 35032740

[33] Al-Shuhaib, M. B. S., Hashim, H. O., & Al-Shuhaib, J. M. B. (2022). Epicatechin is a promising novel inhibitor of SARS-CoV-2 entry by disrupting interactions between angiotensin-converting enzyme type 2 and the viral receptor binding domain: A computational/simulation study. *Computers in Biology and Medicine*, *141*. 10.1016/j.compbiomed.2021.105155 PMC8679518 34942397

[34] Dejani, N. N., Elshabrawy, H. A., & Bezerra Filho, C. D. S. M. (2021). de Sousa, Anticoronavirus and immunomodulatory phenolic compounds: Opportunities and pharmacotherapeutic perspectives. *Biomolecules*, *11*(8). 10.3390/biom11081254 PMC8394099 34439920

[35] Sakshi, C., Harikrishnan, A., Jayaraman, S., Choudhury, A. R., & Veena, V. (2022). Predictive medicinal metabolites from Momordica dioica against comorbidity related proteins of SARS-CoV-2 infections. *Journal of Biomolecular Structure and Dynamics*, *40*(11), 5175–5188. 10.1080/07391102.2020.1868340 33427588 PMC7814569

[36] Mouffouk, C., Mouffouk, S., Mouffouk, S., Hambaba, L., & Haba, H. (2021). Flavonols as potential antiviral drugs targeting SARS-CoV-2 proteases (3CLpro and PLpro), spike protein, RNA-dependent RNA polymerase (RdRp) and angiotensin-converting enzyme II receptor (ACE2). *European Journal of Pharmacology*, *891*. 10.1016/j.ejphar.2020.173759 PMC7691142 33249077

[37] Burley, S. K., Bhikadiya, C., Bi, C., Bittrich, S., Chao, H., Chen, L., Craig, P. A., Crichlow, G. V., Dalenberg, K., Duarte, J. M., & RCSB Protein Data Bank (RCSB. (2023). org): delivery of experimentally-determined PDB structures alongside one million computed structure models of proteins from artificial intelligence/machine learning. *Nucleic Acids Research*, *51*(D1), D488–D508. 36420884 10.1093/nar/gkac1077 PMC9825554

[38] Meng, E. C., Goddard, T. D., Pettersen, E. F., Couch, G. S., Pearson, Z. J., Morris, J. H., & Ferrin, T. E. (2023). UCSF ChimeraX: Tools for structure building and analysis. *Protein Science*, *32*(11), e4792. 37774136 10.1002/pro.4792 PMC10588335

[39] Pettersen, E. F., Goddard, T. D., Huang, C. C., Meng, E. C., Couch, G. S., Croll, T. I., Morris, J. H., & Ferrin, T. E. (2021). UCSF ChimeraX: Structure visualization for researchers, educators, and developers. *Protein science*, *30*(1), 70–82. 32881101 10.1002/pro.3943 PMC7737788

[40] Trott, O., & Olson, A. J. (2010). AutoDock Vina: improving the speed and accuracy of docking with a new scoring function, efficient optimization, and multithreading. *Journal of computational chemistry*, *31*(2), 455–461. 19499576 10.1002/jcc.21334 PMC3041641

[41] Paggi, J. M., Pandit, A., & Dror, R. O. (2024). The art and science of molecular docking. *Annual Review of Biochemistry*, *93*, 389–410. 10.1146/annurev-biochem-030222-120000 38594926 PMC13198409

[42] Baroroh, U., Biotek, M., Muscifa, Z. S., Destiarani, W., Rohmatullah, F. G., & Yusuf, M. (2023). Molecular interaction analysis and visualization of protein-ligand docking using Biovia Discovery Studio Visualizer. *Indonesian Journal of Computational Biology (IJCB)*, *2*(1), 22–30.

[43] Ugbaja, S. C., Mtambo, S. E., Mushebenge, A. G., Appiah-Kubi, P., Abubakar, B. H., Ntuli, M. L., & Kumalo, H. M. (2022). Structural Investigations and Binding Mechanisms of Oseltamivir Drug Resistance Conferred by the E119V Mutation in Influenza H7N9 Virus. *Molecules*, *27*(14). 10.3390/molecules27144376 PMC9317591 35889251

[44] Akinpelu, O. I., Lawal, M. M., Kumalo, H. M., & Mhlongo, N. N. (2020). Drug repurposing: Fusidic acid as a potential inhibitor of M. tuberculosis FtsZ polymerization – Insight from DFT calculations, molecular docking and molecular dynamics simulations. *Tuberculosis*, *121*, 101920. 10.1016/j.tube.2020.101920 32279872

[45] Sinyani, A., Idowu, K., Shunmugam, L., Kumalo, H. M., & Khan, R. (2023). A molecular dynamics perspective into estrogen receptor inhibition by selective flavonoids as alternative therapeutic options. *Journal of Biomolecular Structure and Dynamics*, *41*(9), 4093–4105. 35477414 10.1080/07391102.2022.2062786

[46] Hollingsworth, S. A., & Dror, R. O. (2018). Molecular dynamics simulation for all. *Neuron*, *99*(6), 1129–1143. 30236283 10.1016/j.neuron.2018.08.011 PMC6209097

[47] Zhao, Y., Gee, R., & Doherty, M. F. (2023). Morphology prediction for organic molecular crystals using various force fields in ADDICT. *AIChE Journal*, *69*(8), e18103.

[48] Shunmugam, L., & Soliman, M. E. S. (2018). Targeting HCV polymerase: a structural and dynamic perspective into the mechanism of selective covalent inhibition. *RSC advances*, *8*(73), 42210–42222. 35558797 10.1039/c8ra07346e PMC9092151

[49] Mtambo, S. E., & Kumalo, H. M. (2022). In Silico Drug Repurposing of FDA-Approved Drugs Highlighting Promacta as a Potential Inhibitor of H7N9 Influenza Virus. *Molecules*, *27*(14), 4515. 35889388 10.3390/molecules27144515 PMC9321947

[50] Wells, B. A., & Chaffee, A. L. (2015). Ewald summation for molecular simulations. *Journal of chemical theory and computation*, *11*(8), 3684–3695. 26574452 10.1021/acs.jctc.5b00093

[51] da Fonseca, A. M., Caluaco, B. J., Madureira, J. M. C., Cabongo, S. Q., Gaieta, E. M., Djata, F., Colares, R. P., Neto, M. M., Fernandes, C. F. C., & Marinho, G. S. (2024). Screening of potential inhibitors targeting the main protease structure of SARS-CoV-2 via molecular docking, and approach with molecular dynamics, RMSD, RMSF, H-bond, SASA and MMGBSA. *Molecular Biotechnology*, *66*(8), 1919–1933. 37490200 10.1007/s12033-023-00831-x

[52] Akinpelu, O. I., Lawal, M. M., Kumalo, H. M., & Mhlongo, N. N. (2020). Drug repurposing: Fusidic acid as a potential inhibitor of M. tuberculosis FtsZ polymerization–Insight from DFT calculations, molecular docking and molecular dynamics simulations. *Tuberculosis*, *121*, 101920. 32279872 10.1016/j.tube.2020.101920

[53] Zaib, S., Rana, N., Ali, H. S., ur Rehman, M., Awwad, N. S., Ibrahium, H. A., & Khan, I. (2025). Identification of potential inhibitors targeting yellow fever virus helicase through ligand and structure-based computational studies. *Journal of Biomolecular Structure and Dynamics*, *43*(6), 3031–3048. 38109183 10.1080/07391102.2023.2294839

[54] Chaudhary, N., & Aparoy, P. (2017). Deciphering the mechanism behind the varied binding activities of COXIBs through Molecular Dynamic Simulations, MM-PBSA binding energy calculations and per-residue energy decomposition studies. *Journal of Biomolecular Structure and Dynamics*, *35*(4), 868–882. 26982261 10.1080/07391102.2016.1165736

[55] Chaudhary, N., & Aparoy, P. (2020). Application of per-residue energy decomposition to identify the set of amino acids critical for in silico prediction of COX-2 inhibitory activity. *Heliyon**6*(10), e04944. 10.1016/j.heliyon.2020.e04944 PMC7550918 33083581

[56] Rhman, M. A., Devnarain, N., Khan, R., & Owira, P. M. O. (2022). Synergism potentiates oxidative antiproliferative effects of naringenin and quercetin in MCF-7 breast cancer cells. *Nutrients*, *14*(16), 3437. 36014942 10.3390/nu14163437 PMC9412616

[57] Luo, G., Guenthner, T., Gan, L. S., & Humphreys, W. G. (2004). CYP3A4 induction by xenobiotics: biochemistry, experimental methods and impact on drug discovery and development. *Current drug metabolism*, *5*(6), 483–505. 15578943 10.2174/1389200043335397

[58] Klyushova, L. S., Perepechaeva, M. L., & Grishanova, A. Y. (2022). The role of CYP3A in health and disease. *Biomedicines*, *10*(11), 2686. 36359206 10.3390/biomedicines10112686 PMC9687714

[59] Nozhat, Z., Khalaji, M. S., Hedayati, M., & Kia, S. K. (2022). Different methods for cell viability and proliferation assay: essential tools in pharmaceutical studies, Anti-Cancer Agents in Medicinal Chemistry (Formerly Current Medicinal Chemistry-Anti. *-Cancer Agents)*, *22*(4), 703–712. 10.2174/1871520621999201230202614 33390140

[60] Morse, P. T., Arroum, T., Wan, J., Pham, L., Vaishnav, A., Bell, J., Pavelich, L., Malek, M. H., Sanderson, T. H., & Edwards, B. F. P. (2024). Phosphorylations and acetylations of cytochrome c control mitochondrial respiration, mitochondrial membrane potential, energy, ROS, and apoptosis. *Cells*, *13*(6), 493. 38534337 10.3390/cells13060493 PMC10969761

[61] Muhammed, M. T., & Aki-Yalcin, E. (2024). Molecular docking: principles, advances, and its applications in drug discovery. *Letters in Drug Design & Discovery*, *21*(3), 480–495.

[62] Huang, S. Y., Grinter, S. Z., & Zou, X. (2010). Scoring functions and their evaluation methods for protein–ligand docking: recent advances and future directions. *Physical Chemistry Chemical Physics*, *12*(40), 12899–12908. 20730182 10.1039/c0cp00151a PMC11103779

[63] Shivanika, C., Deepak Kumar, S., Ragunathan, V., Tiwari, P., Sumitha, A., Brindha, P., & Devi (2022). Molecular docking, validation, dynamics simulations, and pharmacokinetic prediction of natural compounds against the SARS-CoV-2 main-protease. *Journal of Biomolecular Structure and Dynamics*, *40*(2), 585–611. 10.1080/07391102.2020.1815584 32897178 PMC7573242

[64] Aliakbar Tehrani, Z., Rulíšek, L., & Černý, J. (2022). Molecular dynamics simulations provide structural insight into binding of cyclic dinucleotides to human STING protein. *Journal of Biomolecular Structure and Dynamics*, *40*(20), 10250–10264. 34187319 10.1080/07391102.2021.1942213

[65] Wei, H., & McCammon, J. A. (2024). Structure and dynamics in drug discovery. *npj Drug Discovery*, *1*(1), 1–8. 42380245 10.1038/s44386-024-00001-2 PMC13267109

[66] Lobato, J. C. M., da Silva Arouche, T., Del Nero, J., dos Santos Borges, R., & Neto, A. M. J. C. (2023). Interactions between carbon nanotubes and external structures of SARS-CoV-2 using molecular docking and molecular dynamics. *Journal of Molecular Structure*, *1286*, 135604. 37089815 10.1016/j.molstruc.2023.135604 PMC10111146

[67] Yasuda, I., Endo, K., Yamamoto, E., Hirano, Y., & Yasuoka, K. (2022). Differences in ligand-induced protein dynamics extracted from an unsupervised deep learning approach correlate with protein–ligand binding affinities. *Communications biology*, *5*(1), 481. 35589949 10.1038/s42003-022-03416-7 PMC9120437

[68] Madasu, P. K., & Chandran, T. (2024). Structural insights into the toxicity of type II ribosome inactivating proteins (RIPs): a molecular dynamics study. *Journal of Biomolecular Structure and Dynamics*, 1–12. 10.1080/07391102.2024.2419855 39466135

[69] Frey, P. A. (2001). Strong hydrogen bonding in molecules and enzymatic complexes. *Magnetic Resonance in Chemistry*, *39*(S1), S190–S198.

[70] Sebastiani, F., Baroni, C., Patil, G., Dali, A., Becucci, M., Hofbauer, S., & Smulevich, G. (2023). The Role of the Hydrogen Bond Network in Maintaining Heme Pocket Stability and Protein Function Specificity of C. diphtheriae Coproheme Decarboxylase. *Biomolecules*, *13*(2), 235. 36830604 10.3390/biom13020235 PMC9953210

[71] Wang, D. D., Zhu, M., & Yan, H. (2021). Computationally predicting binding affinity in protein–ligand complexes: free energy-based simulations and machine learning-based scoring functions. *Briefings in bioinformatics*, *22*(3), bbaa107. 32591817 10.1093/bib/bbaa107

[72] Wang, L., Wang, Y., Yu, Y., Liu, D., Zhao, J., & Zhang, L. (2023). Deciphering Selectivity Mechanism of BRD9 and TAF1 (2) toward Inhibitors Based on Multiple Short Molecular Dynamics Simulations and MM-GBSA Calculations. *Molecules*, *28*(6), 2583. 36985555 10.3390/molecules28062583 PMC10052767

[73] Baron, R., & McCammon, J. A. (2013). Molecular recognition and ligand association. *Annual review of physical chemistry*, *64*(1), 151–175. 23473376 10.1146/annurev-physchem-040412-110047

[74] Velásquez, P. A., Hernandez, J. C., Galeano, E., Hincapié-García, J., & Rugeles, M. T. (2024). W. Zapata-Builes, Effectiveness of Drug Repurposing and Natural Products Against SARS-CoV-2: A Comprehensive Review, Clinical Pharmacology: Advances and Applications 1–25. 10.2147/CPAA.S429064 PMC10773251 38197085

[75] Kaul, R., Paul, P., Kumar, S., Büsselberg, D., Dwivedi, V. D., & Chaari, A. (2021). Promising antiviral activities of natural flavonoids against SARS-CoV-2 targets: systematic review. *International journal of molecular sciences*, *22*(20), 11069. 34681727 10.3390/ijms222011069 PMC8539743

[76] Agrawal, P. K., Agrawal, C., & Blunden, G. (2021). Rutin: A Potential Antiviral for Repurposing as a SARS-CoV-2 Main Protease (M < sup>pro) Inhibitor. *Natural Product Communications*, *16*(4). 10.1177/1934578X21991723

[77] Mohanasundaram, S., Karthikeyan, P., Sampath, V., Anbazhagan, M., Prabhu, S. V., Khaled, J. M., Thiruvengadam, M., & Docking, M. (2024). Dynamics Simulations, ADMET, and DFT Calculations: Combined In Silico Approach to Screen Natural Inhibitors of 3CL and PL Proteases of SARS-CoV-2, Cellular Microbiology (2024) 1–19. 10.1155/2024/6647757

[78] Cherrak, S. A., Merzouk, H., & Mokhtari-Soulimane, N. (2020). Potential bioactive glycosylated flavonoids as SARS-CoV-2 main protease inhibitors: A molecular docking and simulation studies. *Plos One*, *15*(10), e0240653. 33057452 10.1371/journal.pone.0240653 PMC7561147

[79] Xu, Z., Yang, L., Zhang, X., Zhang, Q., Yang, Z., Liu, Y., Wei, S., & Liu, W. (2020). Discovery of potential flavonoid inhibitors against COVID-19 3CL proteinase based on virtual screening strategy. *Frontiers in molecular biosciences*, *7*, 556481. 33134310 10.3389/fmolb.2020.556481 PMC7561382

[80] Fu, Y., Zhao, J., & Chen, Z. (2018). Insights into the molecular mechanisms of protein-ligand interactions by molecular docking and molecular dynamics simulation: A case of oligopeptide binding protein, Computational and mathematical methods in medicine (2018). 10.1155/2018/3502514 PMC6305025 30627209

[81] Caohuy, H., Eidelman, O., Chen, T., Liu, S., Yang, Q., Bera, A., Walton, N. I., Wang, T. T., & Pollard, H. B. (2021). Common cardiac medications potently inhibit ACE2 binding to the SARS-CoV-2 Spike, and block virus penetration and infectivity in human lung cells. *Scientific Reports*, *11*(1), 22195. 34773067 10.1038/s41598-021-01690-9 PMC8589851

[82] Bhowmik, D., Nandi, R., Prakash, A., & Kumar, D. (2021). Evaluation of flavonoids as 2019-nCoV cell entry inhibitor through molecular docking and pharmacological analysis. *Heliyon*, *7*(3), e06515. 10.1016/j.heliyon.2021.e06515 33748510 PMC7955945

[83] Wu, K., Chen, L., Peng, G., Zhou, W., Pennell, C. A., Mansky, L. M., Geraghty, R. J., & Li, F. (2011). A Virus-Binding Hot Spot on Human Angiotensin-Converting Enzyme 2 Is Critical for Binding of Two Different Coronaviruses. *Journal of Virology*, *85*(11), 5331–5337. 10.1128/jvi.02274-10 21411533 PMC3094985

[84] Naresh, G. K. R. S., & Guruprasad, L. (2023). Mutations in the receptor-binding domain of human SARS CoV-2 spike protein increases its affinity to bind human ACE-2 receptor. *Journal of Biomolecular Structure and Dynamics*, *41*(6), 2368–2381. 10.1080/07391102.2022.2032354 35109768

[85] Wang, Z., Huang, W., Zhou, K., Ren, X., & Ding, K. (2022). Targeting the non-catalytic functions: a new paradigm for kinase drug discovery? *Journal of Medicinal Chemistry*, *65*(3), 1735–1748. 35000385 10.1021/acs.jmedchem.1c01978

[86] Raisinghani, N., Alshahrani, M., Gupta, G., & Verkhivker, G. (2024). Ensemble-Based Mutational Profiling and Network Analysis of the SARS-CoV-2 Spike Omicron XBB Lineages for Interactions with the ACE2 Receptor and Antibodies: Cooperation of Binding Hotspots in Mediating Epistatic Couplings Underlies Binding Mechanism and Immune Escape. *International Journal of Molecular Sciences*, *25*(8), 4281. 38673865 10.3390/ijms25084281 PMC11049863

[87] Li, T., Yan, Z., Zhou, W., Liu, Q., Liu, J., & Hua, H. (2024). Discovery of a Potential Allosteric Site in the SARS-CoV-2 Spike Protein and Targeting Allosteric Inhibitor to Stabilize the RBD Down State using a Computational Approach. *Current Computer-Aided Drug Design*, *20*(6), 784–797. 37493168 10.2174/1573409919666230726142418

[88] Alshahrani, M., Gupta, G., Xiao, S., Tao, P., & Verkhivker, G. (2023). Comparative Analysis of Conformational Dynamics and Systematic Characterization of Cryptic Pockets in the SARS-CoV-2 Omicron BA.2, BA.2.75 and XBB.1 Spike Complexes with the ACE2 Host Receptor: Confluence of Binding and Structural Plasticity in Mediating Networks of Conserved Allosteric Sites. *Viruses*, *15*(10). 10.3390/v15102073 PMC10612107 37896850

[89] Kalashnyk, O., Lykhmus, O., Sullivan, R., Komisarenko, S., & Skok, M. (2024). Agonists or positive allosteric modulators of α7 nicotinic acetylcholine receptor prevent interaction of SARS-Cov-2 receptor-binding domain with astrocytoma cells. *Biochemical and Biophysical Research Communications*, *709*, 149825. 38537599 10.1016/j.bbrc.2024.149825

[90] Kumari, M., Singh, R., & Subbarao, N. (2022). Exploring the interaction mechanism between potential inhibitor and multi-target Mur enzymes of mycobacterium tuberculosis using molecular docking, molecular dynamics simulation, principal component analysis, free energy landscape, dynamic cross-correlation matrices, vector movements, and binding free energy calculation. *Journal of Biomolecular Structure and Dynamics*, *40*(24), 13497–13526. 34662260 10.1080/07391102.2021.1989040

[91] Sittel, F., Jain, A., & Stock, G. (2014). Principal component analysis of molecular dynamics: On the use of Cartesian vs. internal coordinates. *The Journal of Chemical Physics*, *141*(1), 014111. 10.1063/1.4885338 25005281

[92] Mani, S., & Swargiary, G. (2023). In Vitro Cytotoxicity Analysis: MTT/XTT, Trypan Blue Exclusion. *Techniques in Life Science and Biomedicine for the Non-Expert*. 10.1007/978-3-031-19485-6_18

[93] Riss, T., Niles, A., Moravec, R., & Karassina, N. (2019). J. Vidugiriene, Cytotoxicity assays: in vitro methods to measure dead cells.

[94] De Jong, W. H., Carraway, J. W., & Geertsma, R. E. In vivo and in vitro testing for the biological safety evaluation of biomaterials and medical devices, Biocompatibility and performance of medical devices, Elsevier2020, pp. 123–166. (Reprinted from.

[95] Szymanski, L., Golaszewska, K., Wiatrowska, A., Dropik, M., Szymanski, P., Gromadka, B., Krakowiak, P., Wierzchowska, J., & Matak, D. (2022). ISO 10993 biological evaluation of novel hemostatic powder – 4SEAL^®^. *Biomaterials Research*, *26*(1). 10.1186/s40824-022-00258-6 PMC8981750 35382888

[96] Hoai, T. T., Yen, P. T., Dao, T. T. B., Long, L. H., Anh, D. X., Minh, L. H., Anh, B. Q., & Thuong, N. T. (2020). Evaluation of the Cytotoxic Effect of Rutin Prenanoemulsion in Lung and Colon Cancer Cell Lines. *Journal of Nanomaterials*, *2020*, 1–11. 10.1155/2020/8867669

[97] Riss,T. L., Moravec, R. A., Niles, A. L., Duellman, S., Benink, H. A., Worzella, T. J., & Minor, L. (2004). Cell Viability Assays. Assay Guidance Manual: Eli Lilly & Company and the National Center for Advancing Translational Sciences: Bethesda,MD, USA. 1–31. http://www.ncbi.nlm.nih.gov/pubmed/23805433

[98] Farhana, A., & Lappin, S. L. Biochemistry, lactate dehydrogenase, Stat Pearls Publishing 2023. (Reprinted from).

[99] Aloufi, A. S., Habotta, O. A., Abdelfattah, M. S., Habib, M. N., Omran, M. M., Ali, S. A., Abdel Moneim, A. E., Korany, S. M., & Alrajhi, A. M. (2023). Resistomycin Suppresses Prostate Cancer Cell Growth by Instigating Oxidative Stress, Mitochondrial Apoptosis, and Cell Cycle Arrest. *Molecules*, *28*(23), 7871. 38067602 10.3390/molecules28237871 PMC10708360

[100] Ye, W., Zheng, Y., Zhang, S., Yan, L., Cheng, H., & Wu, M. (2016). Oxamate Improves Glycemic Control and Insulin Sensitivity via Inhibition of Tissue Lactate Production in db/db Mice. *Plos One*, *11*(3), e0150303. 10.1371/journal.pone.0150303 26938239 PMC4777529

[101] Shen, H. C., Chen, Z. Q., Liu, X. C., Guan, J. F., Xie, D. Z., Li, Y. Y., & Xu, C. (2023). Sodium oxamate reduces lactate production to improve the glucose homeostasis of Micropterus salmoides fed high-carbohydrate diets. *American Journal of Physiology-Regulatory Integrative and Comparative Physiology*, *324*(2), R227–R241. 36572554 10.1152/ajpregu.00226.2022

[102] Zhang, H., & Tsao, R. (2016). Dietary polyphenols, oxidative stress and antioxidant and anti-inflammatory effects. *Current Opinion in Food Science*, *8*, 33–42.

[103] Muscolo, A., Mariateresa, O., Giulio, T., & Mariateresa, R. (2024). Oxidative stress: the role of antioxidant phytochemicals in the prevention and treatment of diseases. *International journal of molecular sciences*, *25*(6), 3264. 38542238 10.3390/ijms25063264 PMC10970659

[104] Zhou, M., Li, J., Xu, J., Zheng, L., & Xu, S. (2023). Exploring human CYP4 enzymes: Physiological roles, function in diseases and focus on inhibitors. *Drug Discovery Today*, *28*(5), 103560. 36958639 10.1016/j.drudis.2023.103560

[105] Mokhosoev, I. M., Astakhov, D. V., Terentiev, A. A., & Moldogazieva, N. T. (2024). Cytochrome P450 monooxygenase systems: Diversity and plasticity for adaptive stress response. *Progress in Biophysics and Molecular Biology*, *193*, 19–34. 10.1016/j.pbiomolbio.2024.09.003 39245215

[106] Nebert, D. W., Wikvall, K., & Miller, W. L. (2013). Human cytochromes P450 in health and disease. *Philosophical Transactions of the Royal Society B: Biological Sciences*, *368*(1612), 20120431. 10.1098/rstb.2012.0431 PMC3538421 23297354

[107] Bai, J., Li, L., Zhao, S., Fan, X., Zhang, J., Hu, M., Chen, Y., Sun, Y., Wang, B., & Jin, J. (2020). Heterotropic activation of flavonoids on cytochrome P450 3A4: A case example of alleviating dronedarone-induced cytotoxicity. *Toxicology Letters*, *319*, 187–196. 31756459 10.1016/j.toxlet.2019.11.016

[108] Kondža, M., Brizić, I., & Jokić, S. (2024). Flavonoids as CYP3A4 Inhibitors In Vitro. *Biomedicines*, *12*(3), 644. 38540257 10.3390/biomedicines12030644 PMC10968035

[109] Alyami, B. A., Zaki, M., Youns, M., Amin, B., Abdou, R., Dawoud, M., & Attia, G. H. (2023). Rutin Inhibits Hepatic and Pancreatic Cancer Cell Proliferation by Inhibiting CYP3A4 and GST. *Ind J Pharm Edu Res*, *57*(2), 1–8.

